# UPR/ATF4/Noxa pathway overactivation through SERCA2 inhibition or ONC201 treatment combined with ABT-737 triggers apoptosis in chemoresistant ovarian cancer cells and patient-derived tumor organoids

**DOI:** 10.1038/s41419-026-08559-7

**Published:** 2026-03-27

**Authors:** Sahra Messaoudi, Romane Florent, Louis-Bastien Weiswald, Guillaume Desmartin, Lucie Lecouflet, Steven Lohard, Léa Grunchec, Edwige Abeilard, Marilyne Guillamin, Jordane Divoux, Benoît Goudergues, Emilie Brotin, Enora Dolivet, Laurent Poulain, Monique N’Diaye

**Affiliations:** 1 Université de Caen Normandie, INSERM, ANTICIPE U1086, Interdisciplinary Research Unit for Cancer Prevention and Treatment, Comprehensive Cancer Center F. Baclesse, Caen, France; 2 UNICANCER, Comprehensive Cancer Center F. Baclesse, Caen, France; 3 Université de Caen Normandie, Services Unit PLATON “Support Platforms for Preclinical and Translational Research in Oncology”, ORGAPRED Core Facility, Caen, France; 4 Université de Caen Normandie, Services Unit PLATON “Support Platforms for Preclinical and Translational Research in Oncology”, ISOCELL, Caen, France; 5 Université de Caen Normandie, Services Unit PLATON “Support Platforms for Preclinical and Translational Research in Oncology”, “OvaRessources” Collection of BioREVA Biological Resource Center, Caen, France; 6 UNICANCER, Comprehensive Cancer Center F. Baclesse, “OvaRessources” Collection of BioREVA Biological Resource Center, Caen, France; 7 Université de Caen Normandie, Services Unit PLATON “Support Platforms for Preclinical and Translational Research in Oncology”, IMPEDANCELL Core Facility, Caen, France; 8 UNICANCER, Comprehensive Cancer Center F. Baclesse, Department of Surgery, Caen, France

**Keywords:** Cancer therapeutic resistance, Apoptosis, Ovarian cancer

## Abstract

Ovarian cancer has a poor clinical prognosis due to chemoresistance following carboplatin/paclitaxel treatment. This phenomenon can be explained by an imbalanced ratio of [anti-apoptotic (BCL-xL/MCL-1)] to [pro-apoptotic (Noxa, BIM, PUMA)] BCL-2 family members that prevents apoptosis initiation. Consequently, any treatment capable of counterbalancing this ratio could be beneficial in the management of ovarian cancer. Calcium signaling is strongly implicated in resistance to apoptosis, as it depends on, but also regulates, the expression of the ratio of BCL-2 family members, making calcium-targeted strategies relevant to overcoming chemoresistance. Knowing that SERCA2 calcium pumps regulation plays a major role in controlling the ER stress-induced UPR response that could lead to pro-apoptotic protein upregulation and cell death, we therefore evaluated whether their inhibition could elicit apoptosis or sensitize ovarian cancer cells to other therapeutic strategies. For this purpose, the platinum-resistant cell line, OAW42-R, was treated with anti-SERCA2 strategies which revert the BCL-2 family member expression ratio in favor of pro-apoptotic proteins. Combination with the BH3-mimetic ABT-737 exacerbates this imbalance not only by inhibiting BCL-xL activity but also through over-induction of the UPR/ATF4/Noxa axis, leading to MCL-1 inhibition. This dual effect of ABT-737 upon ER stress fully suppresses the anti-apoptotic capacities of cancer cells, leading to massive mitochondrial apoptosis. This point was supported with ONC201, the first member of the imipridone family of anticancer drugs to enter the clinic, whose ability to trigger UPR/ATF4/Noxa led to apoptosis commitment when it was combined with ABT-737 treatment. The therapeutic efficacy of these combinations was also proved in patient-derived tumor organoid models (PDTO), leading to their structural disintegration and reduced viability. Collectively, our study highlights that ABT-737, through BCL-xL inhibition and synergy with ER stress inducers, triggers ovarian cancer death, offering promising strategies for overcoming chemoresistance in relapsed ovarian cancer.

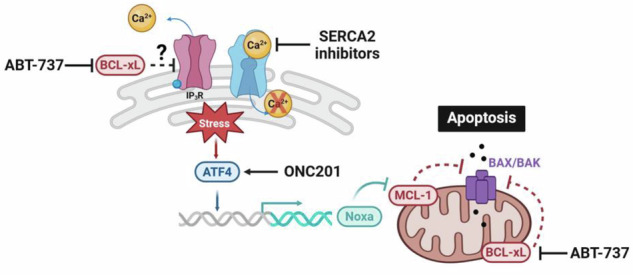

## Introduction

Although ovarian cancer ranks ninth in terms of incidence among women worldwide, it remains the second most deadly form of gynecological cancer in 2022 [1]. Despite a good initial response rate to chemotherapy, 70% of women experience a relapse of the disease, leading to the patient’s death [2]. This chemoresistance is in part due to the occurrence of an adaptative imbalanced [anti-apoptotic (BCL-xL/MCL-1)]/[BH3-only pro-apoptotic (BIM, PUMA, Noxa)] protein ratio, ensuring cancer cells can overcome platinum-triggered pro-apoptotic effects [3–5]. The development of new therapeutic strategies that would counteract this disequilibrium would be a crucial asset to ovarian cancer management. For this purpose, BH3-mimetic molecules (such as ABT-737) that bind to anti-apoptotic proteins to release pro-apoptotic BCL-2 members have demonstrated important results in clinical studies. However, as MCL-1 is not targeted by ABT-737 (which binds BCL-2, BCL-xL, and BCL-W), it remains a hurdle for the full efficacy of this molecule, and strategies aiming to inhibit MCL-1 expression and/or induce its BH3-only protein partners represent relevant approaches to sensitize ovarian carcinoma cells to this BH3-mimetic [6–10].

Targeting calcium signaling has proved to be an interesting path as carcinogenesis is often accompanied by calcium signal modifications to favor cancer cells' proliferation, metabolism, and chemoresistance at the expense of normal cells [11–13]. Moreover, it has been shown that inhibiting calcium flux or Store-Operated Calcium channels impeded Akt/mTOR/MCL-1 axis and sensitized chemoresistant ovarian cancer cells to ABT-737 [14, 15]. In this context, targeting Sarcoendoplasmic Reticulum Calcium ATPase (SERCA) pumps represents another relevant therapeutic strategy [16]. To support this purpose, mipsagargin, a thapsigargin-based pro-agent activated by PSMA-mediated cleavage of an inert masking peptide enabling its vectorization [17], is currently undergoing phase II clinical trials in several cancer sites. SERCA pumps belong to the family of Ca^2+^-ATPases and are encoded by 3 genes (*ATP2A*) leading to SERCA1, 2a, 2b and 3 isoforms. They represent the only transporters that transfer calcium from the cytosol to the endoplasmic reticulum (ER) though ensuring crucial cellular functions as calcium homeostasis control and correct protein folding [18]. Upon SERCA2 inhibition, the dysfunction of calcium-binding proteins involved in protein folding leads to an accumulation of unfolded proteins that compete for binding with the ER stress sensor BiP (Binding-immunoglobulin Protein). This latter dissociates from three proteins, ATF6 (Activating Transcription Factor 6), IRE1α (Inositol-requiring enzyme 1α), and PERK (Protein Kinase R-like ER Kinase)/ATF4, that initiate the Unfolded Protein response (UPR) pathway whose main function consists of reducing global protein synthesis while allowing translation of selected genes to help cellular survival. However, when stress overwhelms the capacity of the adaptive response, the UPR pathway embarks on programmed cell death [18–20].

In light of this mechanism, SERCA2 inhibitors have been shown to trigger cell death or to sensitize cancer cells to various treatments through ER stress activation [21–23]. Many other ER stress inducers displayed interesting results in ovarian cancers [24–26], and among them, the imipridones family of drugs, including ONC201 represent the most relevant and recent molecules for oncological applications. ONC201, also called dordaviprone or TIC10 (TRAIL-Inducing Compound 10), is a first-in-class small molecule that selectively antagonizes the dopamine receptor D2 (DRD2) and hyperactivates ClpP (Caseinolytic proteinase P) that degrades the mitochondrial respiratory chain complex subunits. Downstream of DRD2 and ClpP engagement, ONC201 is well described to activate ATF4/CHOP pathway which leads to TRAIL and its receptor DR5 (Death Receptor 5) upregulation and apoptosis commitment [27]. Pre-clinical studies showed that ONC201 displayed anti-proliferative or cytotoxic effects as a single agent or in combination in several cancer types and is currently being tested in phase 3 clinical trials [27–30].

Considering the strong dependence of ovarian cancer cells on BCL-xL and MCL-1 and the crucial need to find innovative therapeutic combinations to treat ovarian cancer relapses, we evaluated the relevance of strategies inhibiting SERCA2 calcium pumps. Our results showed that impeding SERCA2 expression or activity by RNA interference or by the use of a pharmacological inhibitor imbalanced BCL-2 family expression ratio, priming cells to anti-BCL-xL strategies. However, in addition to its ability to inhibit BCL-xL, ABT-737 strongly enhanced the UPR/ATF4 pathway activated by SERCA2 inhibiting strategies leading to an overexpression of the MCL-1 targeting BH3-only Noxa and a massive cell death. The therapeutic relevance of strategies inducing the UPR/ATF4/Noxa pathway was confirmed by the robust induction of apoptosis following combined treatment with ONC201 and ABT-737. To reinforce the significance of our study, we confirmed our results on highly relevant 3D Patient-Derived Tumor Organoid models (PDTO) supporting promising clinical outcomes in the management of chemoresistant ovarian cancer.

## Materials and methods

### Cell culture

OAW42 cell line is described to come from a human papillary serous cystadenocarcinoma [31] and was obtained from ECACC (Sigma-Aldrich). Chemoresistant sub-line, named OAW42-R, was obtained by exposing cells to increasing doses of cisplatin (Merck) [32]. Cells were cultured in DMEM (Gibco) supplemented with 4500 mg/L glucose, 2 mM GlutamaxTM, 1 mM sodium pyruvate, 33 mM sodium bicarbonate, 20 IU/L recombinant human insulin, and 10% decomplemented bovine serum albumin. OVCAR3 cell line is described to come from a refractory HGSOC (High Grade Serous Ovarian Carcinoma) and was obtained from ATCC. Cells were cultured in RPMI-1460 (Gibco) supplemented with 25 mM HEPES, 4500 mg/L glucose, 33 mM sodium bicarbonate, and 10% decomplemented bovine serum albumin. Cells were maintained at 37 °C in a humid atmosphere enriched with 5% CO_2_.

### Reagents

Thapsigargin was provided by Tocris (R&D Systems), ABT-737 by Selleckem, Z-VAD by Promega and ONC201 by Fisher Scientific. These drugs were stored as stock solutions in DMSO at −20 °C or −80 °C.

### Si-RNA transfection

Non-targeting si-RNA (noted si-CT), si-SERCA2, si-Noxa (si-*PMAIP1*), si-BIM, si-ATF4, si-BAK, si-BAX (ON TARGETplus SMARTpool) were purchased from Dharmacon. Two si-RNA targeting SERCA2 were also used to exclude off-target effects (si-SERCA2-1 sequence: GAAAGUCAAUGUCGGUUUA and si-SERCA2-2 sequence: GGAUCAGAGGUGCUAUUUA). Si-RNA duplexes were diluted in Opti-MEM Reduced Serum Medium (Gibco) and transfected using the INTERFERin^TM^ transfection reagent according to the manufacturer’s instructions (Polyplus-Transfection, France). Cells were treated after a minimum of 24 h of transfection.

### Proliferation analysis

Cell count and viability were estimated using the trypan blue exclusion method.

### Flow cytometry

Annexin V-FITC Kit (Miltenyi Biotec) was used to quantify apoptotic cells according to the manufacturer’s instructions. Briefly, 10^6^ cells were incubated in 1× Binding Buffer containing 10 µL Annexin V-FITC for 15 min. After washing, cell pellet was resuspended in 1× Binding Buffer containing 5 µL of Propidium iodide (PI) immediately prior to analysis by flow cytometry using Cytoflex S (Beckman-Coulter).

### Protein extraction and immunoblotting

#### Protein extraction

Cells were incubated 30 min on ice with lysis buffer: 15 mM HEPES, 50 mM KCl, 10 mM NaCl, 1 mM MgCl_2_, 0.25% glycerol, 0.5% laurylmaltoside, 5 μM GDP, 1 μM microcystin (Enzo Life Sciences), 1 mM sodium orthovanadate, and complete Protease Inhibitor Cocktail (Sigma-Aldrich/Roche). After centrifugation, the supernatant was collected, and proteins were quantified using the Bradford assay (Bio-Rad).

#### Immunoblotting

Equal amounts of proteins were loaded on 4–15% SDS-PAGE precast gel and transferred onto a PDVF membrane (Bio-Rad). Membranes were blocked 1 h with 5% nonfat dry milk in 0.05% Tween-TBS 1× then incubated with the following primary antibodies: anti-ATF4 (#11815), anti-BAK (#3814), anti-BAX (#2774), anti-BCL-xL (#2764), anti-BIM (#2819), anti-BiP (#3177), anti-CASPASE 3 (#9662), anti-CASPASE 8 (#9496), anti-CHOP (#2895), anti-DR5 (#8074), anti-MCL-1 (#5453), anti-PARP (#9542), anti-PUMA (#12450) (Cell Signaling Technology); anti-Noxa (#114C307) (Calbiochem); anti-Actin (#MAB1501) (Merck Millipore); anti-Tubulin (#T6199-100UL) (DM-labo) and anti-SERCA2 (#NB300-581) (Novus-Bio). After incubation with the appropriate horseradish peroxidase-conjugate anti-mouse (#NA931V) (Amersham) or anti-rabbit (#7074) (Cell Signaling Technology), revelation was performed using Clarity Western EleCtroLuminescence detection reagent (Bio-Rad), and signal was recorded using ImageQuant800 (Cytiva).

### RNA extraction, reverse transcription (RT), and quantitative PCR

Total RNAs were isolated using Trizol (Invitrogen, Life Technologies) and quantified using the NanoDrop™ 2000 spectrophotometer (ThermoScientific). RT was realized with the Omniscript reverse transcriptase kit (Qiagen) with random hexamers on a Mastercycler X40 (Eppendorf). For Taq-Man qPCR, cDNA was combined with forward and reverse primers, 1× of the Taq-Man® probe, and Taq-Man® Fast Universal PCR Master Mix (Applied Biosystems). Corresponding custom inventoried (ID: Noxa HS00560402_m1 and GAPDH HS99999905_m1) Taq-Man® Gene Expression Assays were used (Applied Biosystems). For SYBR qPCR, cDNA was combined with MIX 2X (Light Cycler®480 SYBR® Green I Master, Roche) and each forward and reverse primer (Eurogentec):

ATF4-F: 5’-atgaccgaaatgagcttcctg-3’ ATF4-R: 5’-gctggagaacccatgagg-3’

ATP2A1-F: 5’-ctatgagaaggtcggcgagg-3’ ATP2A1-R: 5’-tcattagctggcggatcacc-3’ ATP2A2-F: 5’-tgacaatggcgctctctgtt-3’ ATP2A2-R: 5’-gagccagatgttctcccagg-3’

ATP2A3-F: 5’-ctcccggctgtcatcactac-3’ ATP2A3-R: 5’-cgtcttgtcggagcagatga-3’

GAPDH-F: 5’-gaaagcctgccggtgactaa-3’ GAPDH-R: 5’-aggaaaagcatcacccggag-3’.

All PCR reactions were carried out on a LightCycler 480 (Roche). GAPDH was used as a housekeeping gene for normalization, and relative fold changes in expression were calculated using the 2^-ΔΔCq^ method.

### PDTO establishment, culture, and treatments

#### Patients samples

PDTOs were generated by culturing cancer cells obtained through dissociation of surgical specimens of chemonaive ovarian tumors (OV-253-T) or ascites (OV-130-A) from patients with HGSOC at the Comprehensive Cancer Center Francois Baclesse (Unicancer Center, Normandy). Cancer histology was confirmed by a pathologist. Written informed consent was obtained for the subject and the study was approved by the North West III’ ethical committee (IDRCB: 2018-A02152-53). PDTO histological and genomic characters as well as their chemoresistance assessment were described in Thorel et al. [33].

#### PDTO establishment

PDTOs are obtained as previously described [34].

#### PDTO treatments and viability assay

PDTO with a diameter of around 150 µm were collected in cold Organoid Basal Medium (Advanced DMEM, 10 UI/mL penicillin, 10 µg/mL streptomycin, 1% GlutaMAX-1 (Fisher Scientific)) supplemented with 1% BSA and centrifugated at 200× *g* for 2 min. PDTO were resuspended in organoid treatment medium and counted. Fifty PDTO were seeded per well in 20 μL of 10% Basement Membrane Extract type 2 (BME2)/organoid treatment medium in a black clear-bottom 384-well plate (Greiner). Twenty µL of 2X-treatment was then added to the same BME2/organoid treatment medium and PDTO were incubated for an appropriated time. CellTiter-Glo assay was performed according to the manufacturer’s instruction and luminescence was measured using GloMax Discover Microplate Reader (Promega). Results were normalized to vehicle and represented as the average of three independent biological replicates.

### Statistical analysis

All statistical analyses and graphs were generated with GraphPad Prism 8.1. The values were presented as means ± S.D. for at least three independent experiments. *T* test was used for statistical analysis. Results were considered statistically different if *P* < 0.05 (*); *P* < 0.01 (**); *P* < 0.001 (***); *P* < 0.0001(****).

## Results

### SERCA2 targeting strategies induce BH3-only proteins in OAW42-R cell line

We analyzed the expression of SERCA isoforms in the OAW42-R cell line which exhibits acquired chemoresistance induced by multiple cisplatin treatments that mimics the most common clinical situation. It appears that *ATP2A2* (gene encoding SERCA2) is around 500 times more expressed than *ATP2A1* and around 6500 times more expressed than *ATP2A3*, supporting that SERCA2 is the dominant isoform in OAW42-R cells (Supplementary Fig. S1A). A transfection kinetic was performed and revealed that SERCA2 protein expression is decreased as early as 24 h with a maximum at 72 h of transfection, suggesting that SERCA2 has a long protein half-life as observed elsewhere [35] (Supplementary Fig. S1B). This extinction was also specific to *ATP2A2* as it did strongly modify *ATP2A1* and *ATP2A3* mRNA expression (Supplementary Fig. S1C).

**Fig. 1:**
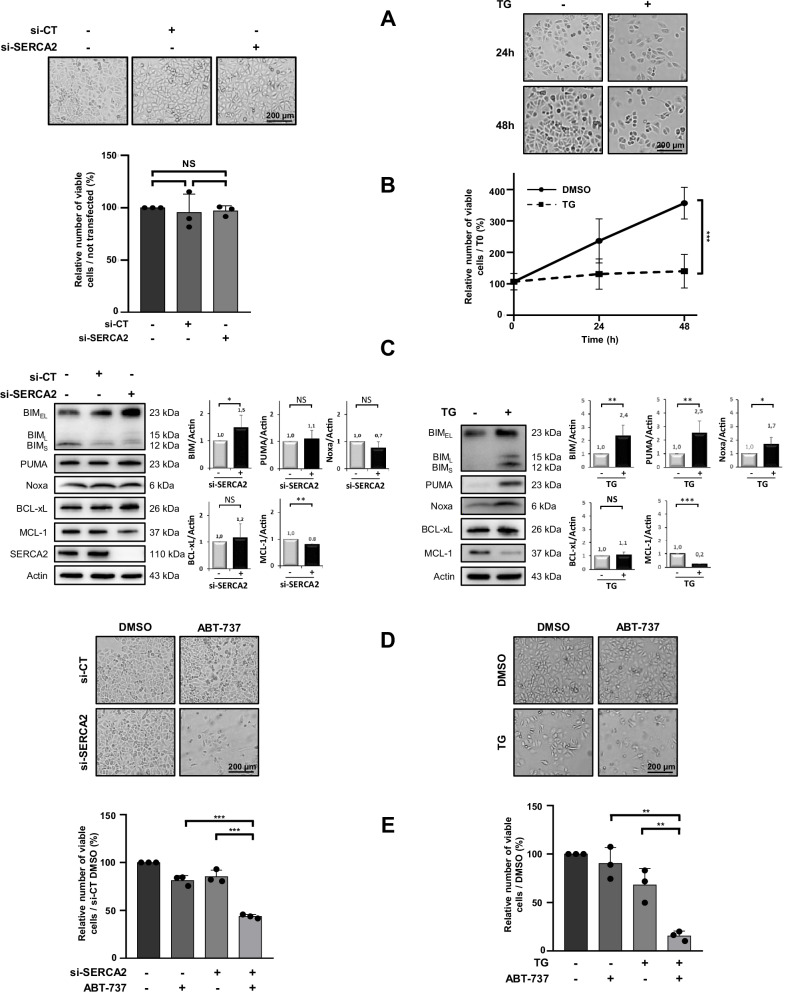
SERCA2 targeting with si-RNA and thapsigargin induces BH3-only protein expression and sensitizes OAW42-R cell line to ABT-737. Chemoresistant OAW42-R cells were either transfected 72 h with 20 nM si-control (si-CT) or si-SERCA2 (left column) or treated with 0,5 µM thapsigargin (TG) for 24 h and 48 h (right column) and the effects were investigated **A** on cell morphology by optical microscopy (scale bar = 200 µM), **B** on cell proliferation by the Trypan blue exclusion test (histograms represent the percentage of viable cells in the transfected conditions normalized to that of no transfected one (100%) (left column) and in the treated condition normalized to that of T0 condition (100%) (right column)), and **C** on the BCL-2 family members expression using western blot analysis. Protein expression levels were quantified using ImageJ software and normalized to that of actin for the si-CT or DMSO. OAW42-R were transfected with 20 nM si-CT or si-SERCA2 for 72 h followed by a 24 h-treatment with 5 µM ABT-737 (left column) or co-treated with 0.5 µM thapsigargin and 5 µM ABT-737 for 24 h (right column) and their effects were assessed on **D** cell morphology by optical microscopy (scale bar = 200 µM) and on **E** cell proliferation by the Trypan blue exclusion test (histograms represent the percentage of viable cells in each conditions normalized to that of the control condition (100%)) (*N* = 3 for all experiments). Results were considered statistically different if *P < 0.05, **P < 0.01, ***P < 0.001.

Optical microscopy and cell counting showed that si-SERCA2 did not impact cell proliferation and viability (Fig. 1A, B, left). As for its effect on BCL-2 family members, it led to an increase in the pro-apoptotic members BIM, had no effect on BCL-xL and slightly decreased MCL-1 expression (Fig. 1C, left). We also used the pharmacological inhibitor thapsigargin to abrogate the activity of SERCA2 during 24 and 48 h. Unlike si-SERCA2, thapsigargin (TG) displayed an anti-proliferative action, but no cytotoxic effect was observed based on morphological observations (Fig. 1A, B, right). Concerning its effect on BCL-2 family members, thapsigargin increased BIM, PUMA as well as Noxa after 24 h of treatment (Fig. 1C, right), had no effect on BCL-xL while inhibiting MCL-1 protein expression (Fig. 1C, right). Collectively, both SERCA2 targeting strategies induced the expression of BH3-only proteins and inhibited the expression of MCL-1, leading to an imbalanced [pro-apoptotic]/[anti-apoptotic] protein ratio.

### SERCA2 targeting strategies sensitize OAW42-R cell line to ABT-737

As they deregulate BCL-2 family members expression ratio, si-SERCA2 and thapsigargin could be candidates to sensitize OAW42-R cells to ABT-737. Cells were either transfected for 72 h with si-SERCA2 before being treated with ABT-737 for 24 h or co-treated with thapsigargin and ABT-737 for 24 h. Whereas ABT-737 alone did not have any effect on its own, its combination with si-SERCA2 or thapsigargin triggered a massive cell death as observed by microscopy and cell counting (Fig. 1D, E), confirming the cytotoxic effect of both combinations.

### SERCA2 targeting strategies combined with ABT-737 lead to mitochondrial apoptosis

**Fig. 2:**
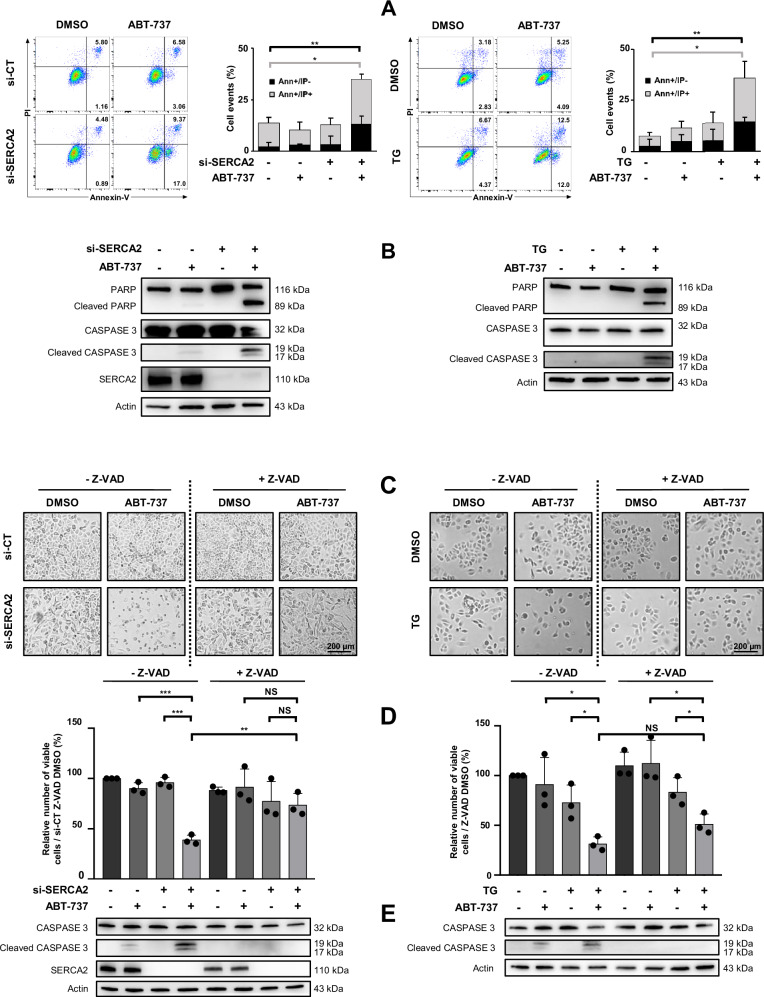
SERCA2 targeting sensitizes OAW42-R cell line to ABT-737 by inducing apoptosis. OAW42-R were transfected with 20 nM si-CT or si-SERCA2 for 72 h followed by a 10 h-treatment with 5 µM ABT-737 (left column) or co-treated with 0.5 µM thapsigargin and 5 µM ABT-737 for 6 h (right column). Apoptosis involvement was investigated, **A** by measuring Annexin V/FITC events using flow cytometry and, **B** by analyzing PARP and CASPASE 3 cleavages by western blot (*N* = 3). Effects of 50 µM Z-VAD were investigated in OAW42-R cells. Cells were either transfected 72 h with si-SERCA2 then pre-treated with Z-VAD for 1 h and 30 min and finally treated with ABT-737 for 24 h, or pre-treated with Z-VAD for 1 h 30 min and treated with 0.5 µM thapsigargin + 5 µM ABT-737 for 24 h. Apoptosis involvement was investigated **C** on cell morphology by optical microscopy (scale bar = 200 µM), **D** on cell viability by the Trypan blue exclusion test (histograms represent the percentage of viable cells normalized to that of the control condition (100%)) and, **E** by the observation of CASPASE 3 cleavage by western blot (*N* = 3). Results were considered statistically different if *P < 0.05, **P < 0.01, ***P < 0.001.

In order to characterize the nature of cell death, Annexin V analysis by flow cytometry has been performed. Whereas si-SERCA2, thapsigargin or ABT-737 used as single agents only triggered modest apoptosis, a significant increase in the population of early (Annexin V + /IP-) and late (Annexin V + /IP + ) apoptotic cells were observed following exposure to both combinations (Fig. 2A). Apoptotic cell death was confirmed by the appearance of PARP and CASPASE 3 cleavages (Fig. 2B) and the capacity of Z-VAD, to impede cell detachment (Fig. 2C), cell death (Fig. 2D) and CASPASE 3 cleavage (Fig. 2E) supporting the protective effect of this inhibitor (although to a lesser extent for thapsigargin/ABT-737) and CASPASE involvement in the cytotoxic effect of both combinations. Evaluation of BAX and BAK protein expression upon treatments revealed that these proteins were not modulated, regardless of the condition studied (Supplementary Fig. S2A). Moreover, their silencing by si-RNA precluded cell death (Supplementary Fig. S2B–D), supporting the involvement of Mitochondrial Outer Membrane Permeabilization (MOMP) in OAW42-R cell death.

### Noxa is the key regulator of SERCA2 targeting strategies/ABT-737-induced apoptosis

**Fig. 3:**
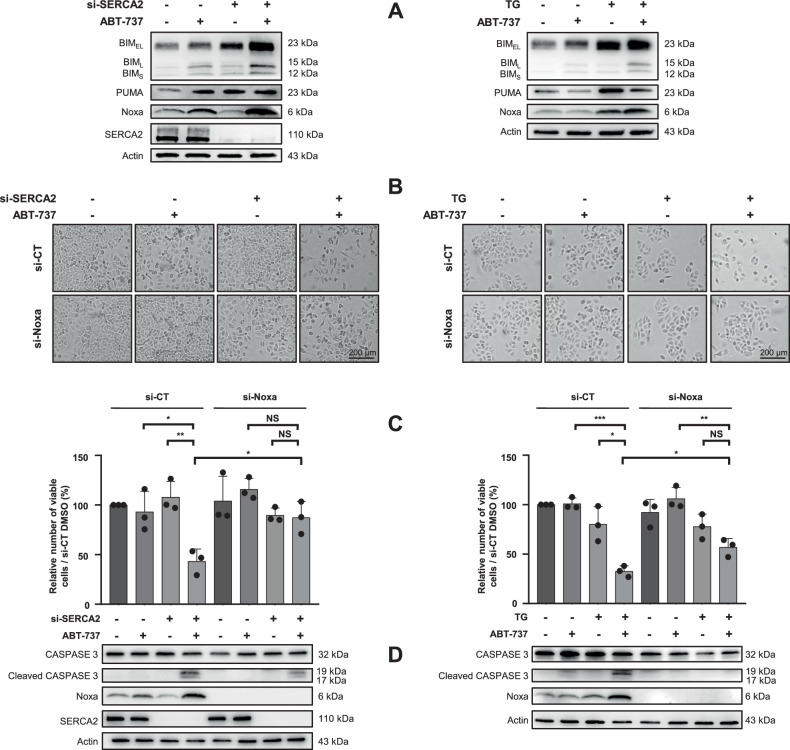
SERCA2 targeting strategies synergize with ABT-737 treatment, leading to Noxa overexpression that triggers apoptosis. **A** Pro-apoptotic BIM, PUMA, and Noxa protein expression were investigated by western blot after both combinations in OAW42-R cells (*N* = 3). Cells were co-transfected with 10 nM si-Noxa and 10 nM si-SERCA2 for 72 h then treated with 5 µM ABT-737 for 24 h (left column) (*N* = 3) or transfected 24 h with 20 nM si-Noxa, then treated 24 h with 0.5 µM thapsigargin + 5 µM ABT‑737 (right column) (*N* = 4). Cell death was investigated on **B** cell morphology by optical microscopy (scale bar = 200 µM), **C** cell viability by the Trypan blue exclusion test (histograms represent the percentage of viable cells normalized to that of the control condition (100%)), and on **D** the expression of CASPASE 3 cleavage by western blot. Results were considered statistically different if *P < 0.05, **P < 0.01, ***P < 0.001.

To determine which pro-apoptotic member was involved in apoptosis, we studied BH3-only protein expression upon co-treatments. Results revealed that, on the one hand, ABT‑737 increased basal PUMA expression and did not modulate the effect of si-SERCA2 on PUMA (Fig. 3A, left panel). On the other hand, ABT-737 decreased both basal and thapsigargin-induced PUMA expression, suggesting PUMA was not fully involved in cell death (Fig. 3A, right panel). Regarding BIM and Noxa, ABT-737 triggered their over-induction when combined with si-SERCA2 or thapsigargin. Knockdown of these BH3-only proteins revealed that whereas BIM silencing had no protective effect (data not shown), Noxa silencing was sufficient to preclude apoptosis (Fig. 3B–D), supporting its pivotal role in both combinations-induced cytotoxicity.

### Noxa expression is increased through UPR pathway and ATF4

**Fig. 4:**
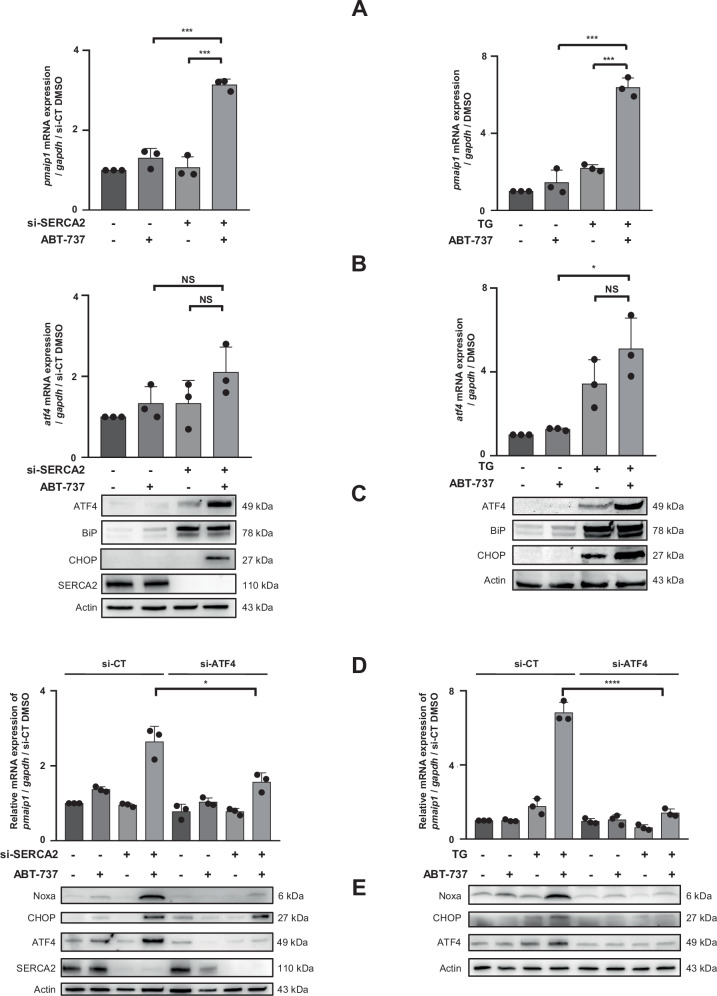
Noxa protein increase is controlled by ER stress-induced ATF4 transcription factor. **A** Expression of *PMAIP1* mRNA (coding Noxa protein) was investigated by RT-qPCR after both combinations in OAW42-R cells (si-SERCA2 + ABT-737: left column and thapsigargin+ABT‑737: right column). **B** ATF4 mRNA expression was evaluated by RT-qPCR and **C** expression of ER stress markers BiP, ATF4, and CHOP were assessed by western blot. After 72 h-transfection with 20 nM si-SERCA2, cells were transfected 24 h with 20 nM si-ATF4 followed by a 24 h-treatment with 5 µM ABT-737 (left column) or transfected with 20 nM si-ATF4 followed by a 24 h-treatment with 0.5 µM thapsigargin + 5 µM ABT-737 (right column). **D** *PMAIP1* mRNA expression was analyzed by RT-qPCR and **E** Noxa, CHOP and ATF4 protein expression were assessed by western blot. Results were considered statistically different if *P < 0.05, **P < 0.01, ***P < 0.001, ****P < 0.0001 (*N* = 3 for all experiments).

Quantitative PCR was then performed to decipher the mechanisms underlying Noxa protein increase. Whereas neither ABT-737 nor si-SERCA2 induced *PMAIP1* expression, thapsigargin doubled it, and both combination conditions led to *PMAIP1* transcription over-induction (x3.1 and x6.4, respectively) (Fig. 4A). As inhibition of SERCA2 calcium pumps could trigger ER stress and UPR-induced BH3-only proteins transcription, expression of the ER stress markers BiP, ATF4, and CHOP was evaluated. Although ABT-737 did not have any effect on its own, it strongly reinforced BiP, ATF4 and CHOP protein expression induced by si-SERCA2 and thapsigargin supporting a robust UPR triggering in the combination conditions. In addition, both ABT-737 and si-SERCA2 slightly up-regulated ATF4 transcription (x1.3) and the combination potentiated this effect (x2.1) while thapsigargin elicited a strong increase on its own (x3.4) leading to a more potent ATF4 transcription when combined with ABT-737 (x5.1) (Fig. 4B, C). Taken together, ABT-737 and si-SERCA2 used as single agents could slightly increase *ATF4* transcription, which was not potent enough to induce a strong ATF4 protein expression and Noxa upregulation. However, the threshold is overwhelmed when they are combined. As for thapsigargin, it could trigger transcriptional and translational ATF4 and Noxa inductions on its own, an effect that is boosted by its combination with ABT-737. Finally, ATF4 silencing effectively inhibited Noxa mRNA and protein induced by both combinations, comforting the central role of ATF4 in Noxa upregulation in OAW42-R cells (Fig. 4D, E and Supplementary Fig. S3). As CHOP is described to be a transcriptional target of ATF4 as well as to cooperate with it in some models, we tested its involvement in Noxa induction. Results showed that si-ATF4 did not abrogate CHOP protein induction (Fig. 4E), and silencing CHOP did not inhibit Noxa expression as observed elsewhere [36, 37] (Supplementary Fig. S4).

These results were also obtained with 2 deconvoluted si-RNA targeting SERCA2, which rules out off-target effects (Supplementary Fig. S5A–C).

### ONC201 increases BH3-only proteins expression and sensitizes OAW42-R cell line to ABT-737 through ATF4/Noxa induction

**Fig. 5:**
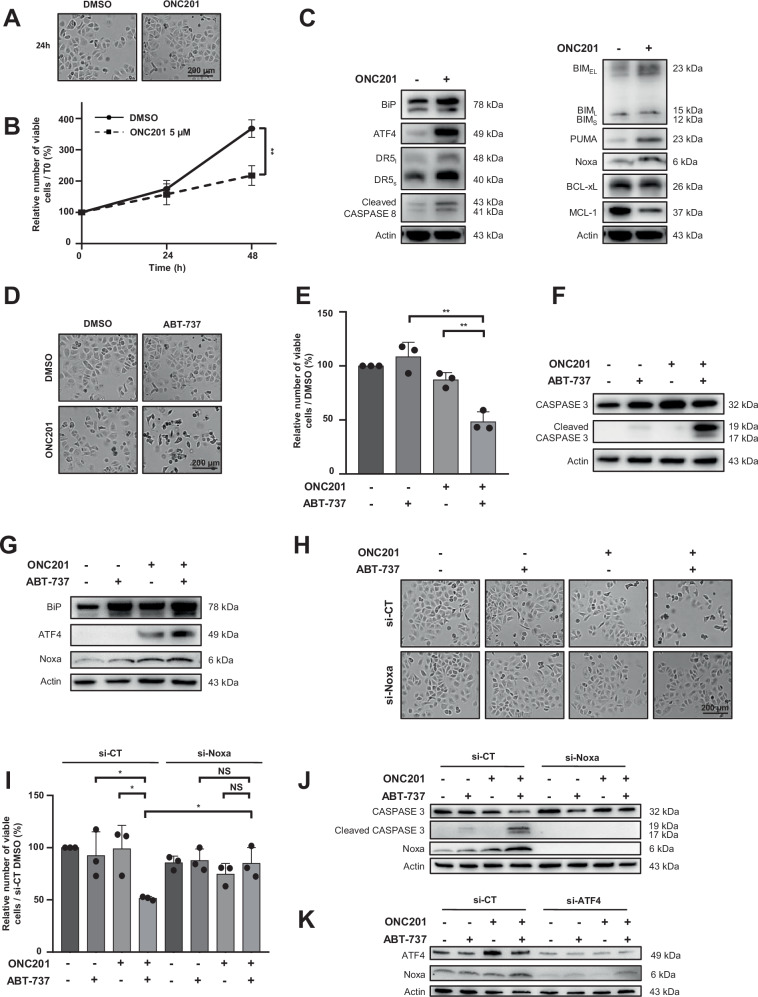
ONC201 synergizes with ABT-737 to induce apoptosis through ER stress/ATF4/Noxa pathway. Effects of 5 µM ONC201 were investigated in OAW42-R cells after 24 and 48 h treatment **A** on cell morphology by optical microscopy (scale bar = 200 µM; 24 h-treatment), **B** on cell proliferation by the Trypan blue exclusion test (histograms represent the percentage of viable cells in the treated conditions normalized to that of T0 condition (100%)). **C** Expression of ONC201 activity markers (BiP, ATF4, DR5, and cleaved CASPASE 8) and BCL-2 family members were assessed by western blot analysis after 24 h treatment with ONC201 5 µM (*N* = 3). Effects of a 24 h-co-treatment with 5 µM ONC201 + 5 µM ABT‑737 were investigated in OAW42-R cells on **D** cell morphology by optical microscopy (scale bar = 200 µM), **E** cell viability assessed by the Trypan blue exclusion test (histograms represent the percentage of viable cells normalized to that of the control condition (100%)), on **F** apoptosis marker expression by analyzing CASPASE 3 cleavage by western blot, and on **G** BiP, ATF4 and Noxa protein expression by western blot (*N* = 3). Cells were transfected with 20 nM si-Noxa for 24 h then treated with 5 µM ONC201 in the presence or in the absence of 5 µM ABT-737. **H** Cell morphology was analyzed by optical microscopy (scale bar = 200 µM), **I** cell proliferation by the Trypan blue exclusion test (histograms represent the percentage of viable cells in the treated conditions normalized to that of the control condition (100%)), and **J** apoptosis triggering was assessed by observation of CASPASE 3 cleavage by western blot (*N* = 3). **K** Cells were transfected with 20 nM si-ATF4 for 24 h then treated with 5 µM ONC201 in the presence or in the absence of 5 µM ABT-737. ATF4 and Noxa expression were assessed by western blot. Results were considered statistically different if *P < 0.05, **P < 0.01, ***P < 0.001.

As potent and selective SERCA2 inhibitors are not currently available for clinical use, we tested if activating downstream events, as ER stress, was a relevant strategy to sensitize to ABT-737. For this purpose, cells were exposed to ONC201 for 24 h and 48 h, a treatment that did not produce any morphological change or cytotoxic effect, although it induced an anti-proliferative effect for 48 h exposure (Fig. 5A, B). We then confirmed that ONC201 was fully effective in our model through the upregulation of its markers of activity ATF4, DR5, and cleaved CASPASE 8 (Fig. 5C) and showed that this compound increased BIM, PUMA, and Noxa, decreased MCL-1 and had no effect on BCL-xL expression (Fig. 5C). Its combination with ABT-737 elicited massive apoptosis, confirming the anti-tumoral efficacy of this therapeutic strategy (Fig. 5D–F). This cell death came along with the induction of ER stress markers BiP, ATF4 but also the BH3-only Noxa (Fig. 5G), whose inhibitions were sufficient to protect cells against ONC201/ABT-737-induced apoptosis (Fig. 5H–K).

All these results were also obtained in the cisplatin-refractory OVCAR3 cell line, whose molecular phenotype is more representative of the HGSOC profile than that of OAW42-R [31, 38]. The OVCAR3 cell line expresses SERCA2, ATF4, and the BCL-2 family members Noxa, MCL-1, BCL-xL, BIM and to a lesser extent PUMA. It should be remarked that OVCAR3 cell line expressed ATF4 at basal level contrarily to OAW42-R cell line, suggesting that ER stress is constitutively activated. Thapsigargin and ONC201 treatment induced ATF4 and Noxa expression while inhibiting MCL-1 (Supplementary Fig. S6A). Combinations triggered OVCAR3 cell line death (in morphological features and counting) which was accompanied with Noxa induction and caspase 3 cleavage. Cell death was efficiently repressed through Noxa silencing confirming the results we obtained with OAW42-R. Surprisingly, ABT-737 seemed to slightly repress ONC201 and thapsigargin-induced ATF4 as already shown elsewhere [39] (Supplementary Fig. S6B–D). However, silencing ATF4 fully inhibited Noxa induction, suggesting that ATF4 controls at least partially Noxa expression in this model (Supplementary Fig. S6 E). Taken together, these results revealed that the molecular mechanisms are similar, regardless of the molecular phenotype or histological subtype of ovarian cancer.

### Thapsigargin and ONC201 sensitize HGSOC-derived organoids to ABT-737

**Fig. 6:**
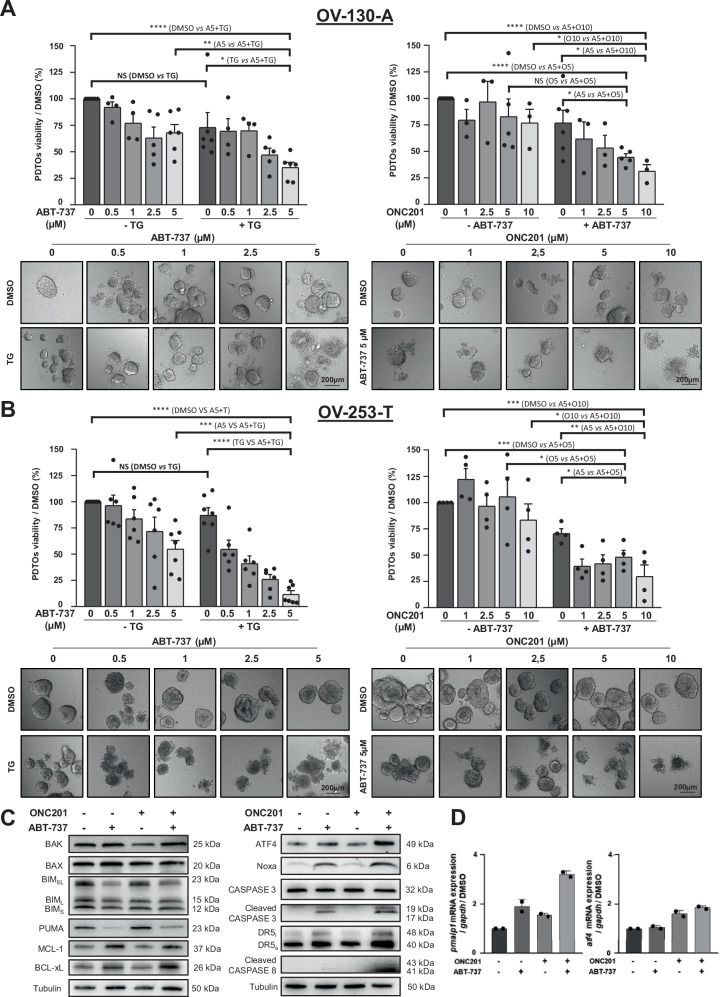
Thapsigargin or ONC201 combined with ABT-737 triggers apoptotic cell death in PDTOs. Two naive PDTOs lines (OV-130-A and OV-253-T) from patients with chemoresistant HGSOC were treated with 0,5 µM thapsigargin and increasing concentrations of ABT-737 for 24 h (left column) or with 5 µM ABT‑737 with increasing concentrations of ONC201 (right column). PDTOs viability was measured in each condition by CellTiter-Glo 3D assay (histograms represent the percentage of PDTO viability in treated conditions normalized to that of the control condition) (upper panels). Morphological effect on PDTO was assessed by optical microscopy (scale bar = 200 μm) (lower panels). **A** Results for OV-130-A PDTOs line (*N* ≥ 4 and *N* ≥ 3), **B** results for OV-253-T PDTOs line (*N* ≥ 6 and *N* = 4). **C** OV-253-T PDTOs line was co-treated with 5 µM ONC201 and 5 µM ABT-737 for 24 h and expression of BCL-2 family members, ONC201 activity markers (DR5, cleaved CASPASE 8), ER stress markers (BiP, ATF4), Noxa and the apoptosis marker (cleaved CASPASE 3) were assessed (*N* = 3) and **D** *pmaip1* and *atf4* mRNA levels were studied by RT-qPCR (*N* = 2).

To evaluate whether SERCA2 inhibitors or ONC201 combined to ABT-737 has an anticancer efficacy in a model closer to clinical situation, we tested their combination on two naive chemoresistant PDTO lines established from ascites (OV-130-A – Fig. 6A) or tumor (OV-253-T – Fig. 6B) of patients with HGSOC [33]. Whereas any compound used as single agent displayed strong cytotoxic effect for both PDTO lines tested, the combinations triggered cell death and a disintegration of these models as soon as 24 h of treatment confirming the clinical relevance of these strategies (Fig. 6A, B). It should be noted that PDTO derived from ascitic fluid seemed more resistant than those derived from the tumor specimen. Analysis of the underlying molecular events indicated that ONC201 combined with ABT-737 did not over-induce any BCL2 family member expression except Noxa, and this co-treatment also triggered ATF4 upregulation and CASPASE 3 cleavage. (Fig. 6C). *Atf4* and *pmaip1* mRNA were also induced by the combination, suggesting a transcriptional pathway. Taken together, thapsigargin and ONC201 combined with ABT-737 triggered significant mortality in PDTO and these latest results strongly suggest that this effect involves the ATF4/Noxa axis highlighting a novel therapeutic approach to overcome chemoresistance in ovarian cancer.

## Discussion

In the present work, we explored whether targeting SERCA calcium pumps, which regulate ER calcium homeostasis and the UPR pathway, could modulate the expression of pro-apoptotic proteins to sensitize ovarian cancer cells to innovative therapeutic strategies.

We found that *ATP2A2* mRNA was the predominant isoform compared to *ATP2A1* and *ATP2A3* in OAW42-R cell line. This result is supported by Bertocci in colorectal cancer [40] and is in agreement with the observation that SERCA2 is ubiquitously found in normal and cancer cells. By contrast, SERCA1 is commonly restricted to skeletal muscle, while SERCA3 is decreased in most types of cancer [23, 35, 41, 42].

Targeting SERCA2 through gene silencing did not affect the proliferation of ovarian cancer cells, a result also observed in HeLa cells [23]. In contrast, thapsigargin effectively inhibited OAW42-R ovarian cancer cell growth, a result that is consistent with findings in breast cancer [43], melanoma [44], and esophageal cancer models [21]. In addition to inhibiting SERCA calcium pumps, thapsigargin can activate the NADPH oxidase system, increase reactive oxygen species (ROS), and deplete glutathione (GSH), mechanisms known to suppress proliferation in ovarian cancer cells [45].

In our experiments, 0.5 µM thapsigargin used for 24 h did not induce cell death as a single agent, which aligns with observations in IGROV1-R10 ovarian cancer cells [46] and adrenocortical carcinoma [47]. Despite the fact that sensitivity to thapsigargin can vary based on cellular context, cell death is often observed with higher concentrations or longer treatment durations [21, 47–50].

This treatment was however sufficient to induce the expression of BH3‑only proteins and to reduce MCL-1 expression which is consistent with literature [46, 51–55]. This effect was partially reproduced with si-SERCA2 which induced BIM and reduced MCL-1 expression. These variations could be attributed to differences in time of treatments and possible side effects. Indeed, in addition to inhibit the SERCA calcium pumps, thapsigargin can dysregulate redox status, a mechanism known to induce BH3-only proteins expression [21, 45, 56]. Collectively, these results highlighted that SERCA2 inhibition leads to an imbalanced [pro-]/[anti-apoptotic] ratio whatever the means employed to inhibit these calcium pumps.

This effect was relevantly used to sensitize the OAW42-R cell line to anti-BCL-xL strategies, and Annexin V/IP analysis, assessment of CASPASE 3 cleavage, and the inhibitory effect of Z-VAD highlighted the apoptotic nature of cell death induced by both combinations. It should be noted that Z-VAD did not completely suppress thapsigargin/ABT-737-induced cell death despite the complete inhibition of CASPASE 3 cleavage, suggesting induction of other types of cell death as depicted elsewhere [57, 58].

Consistent with our previous work and others, ABT-737 induced Noxa and BIM expression [8, 24, 59] as well as enhanced SERCA2 inhibitors effect. As for PUMA, its reduced expression upon ABT-737 treatment was also observed in colon, myeloma and other ovarian cell lines but mechanisms still remain obscure [24, 60, 61]. The protective effect of Noxa gene silencing suggests that Noxa upregulation was potent enough to efficaciously counteract MCL‑1 activity and triggered apoptosis, a result in complete agreement with the pivotal role played by Noxa/MCL-1 axis in ABT-737 resistance [8, 24, 62, 63].

Analysis of molecular pathways showed that Noxa increase depends on transcriptional events involving ER stress and ATF4 as described elsewhere [19, 64–66]. It could be hypothesized that the modest increase in ATF4 transcription induced by ABT-737 or si-SERCA2 was not strong enough to up-regulate ATF4 and Noxa protein expression but was sufficient to prime ER stress and eventually to launch UPR response and Noxa upregulation when they were combined. This purpose is supported by the fact that si-SERCA2 increased BiP, confirming that ER stress is already present, whereas ATF4 protein is not yet fully expressed. Moreover, the sharper ER stress triggered by thapsigargin used as single agent induced the transcription of *ATF4* but also its translation leading to a stronger increase of Noxa.

ABT-737 effect on ER stress could be attributed to its capacity to interfere with ER calcium content. Actually, BCL-xL was described to interact with IP3R (Inositol 1,4,5-trisphosphate Receptor) at the ER to dampen IP3R-dependent Ca^2+^ release, acting thus as an inhibitor of the ER stress response [67]. Moreover, this interaction with IP3R was demonstrated to involved residue K87, located in the BH3 domain of BCL-xL. We thus speculated that ABT-737 binding to BCL-xL unleash IP3R that led to a release of calcium from the ER to the cytosol which not only facilitate calcium re-uptake by the mitochondria favoring apoptosis events but also reduce ER calcium content that triggered ER stress and UPR response [68, 69]. This result could also explain the discrepancies observed with ABT-737 to act or not through *ATF4/PMAIP1* transcriptional induction as, depending on ABT-737 concentrations, duration of treatment and cancer models, the threshold leading to ATF4 activation and its downstream events is not always reached [70–72].

Another approach to mimic SERCA2 inhibition effect is to elicit the downstream pathways induced by the disruption of calcium homeostasis leading to ER stress [73]. For this purpose, we focused on ONC201 potential sensitizing effect as this first-in-class molecule was described to trigger anti-proliferative and cytotoxic effect through activation of the UPR/ATF4 pathway [27, 29, 74]. In our hands, ONC201 mimicked thapsigargin anti-proliferative effect [28] and imbalanced the [anti-]/[pro-anti-apoptotic] ratio, a result supported by others for BIM upregulation [29, 75] and MCL-1 downregulation [28, 29, 76, 77]. These results could be attributed to its capacity to inhibit PI3K/Akt pathway leading to MCL-1 repression and to FOXO3A/BIM upregulation. Regarding PUMA and Noxa, ONC201 strongly increased their expression which differs from previous works displaying either an absence of modulation or a downregulation of these BH3-only proteins [39, 78]. These variations could certainly be attributed to cell line-dependent context and variations in time and dose treatment.

ONC201 induced-imbalanced ratio synergized with BH3-mimetic and led to a massive apoptotic cell death as observed in glioblastoma [78] and in other ovarian cancer cell lines [39]. ONC201/ABT-737 combination led to an overexpression of BIM, and to the best of our knowledge, this is the first time this result has been observed. This combination also induced the overexpression of Noxa, a result that has been inconsistently observed [39], but whose crucial role in apoptosis commitment was demonstrated by the complete rescue of ovarian cancer cells upon Noxa gene silencing, as observed in glioblastoma [78].

ER stress and ATF4 are commonly activated in cancer cells to regulate metabolism and help cells adapt to challenging environments, such as hypoxia, thereby supporting their role in carcinogenesis and chemoresistance. However, when the cellular stress exceeds the cell’s ability to buffer it, apoptosis can occur, and this delicate balance may present a potential therapeutic opportunity [19]. Notably, ATF4 expression was not observed under basal conditions in OAW42-R cells, indicating that ER stress was not inherently activated in these cells. The robust upregulation of ATF4 and Noxa in response to ER stressors and ABT-737 effectively triggers cell death. In contrast, OVCAR3 HGSOC cells express ATF4 at basal levels, suggesting that this cell line depends on ER stress for survival. Nevertheless, thapsigargin and ONC201 induced overexpression of this transcription factor and Noxa. It should be noted that the concentration of thapsigargin used is much lower than that used for OAW42-R, while the concentration of ONC201 used to induce ER stress remained the same. This suggests that this cell line is more sensitive to the SERCA2 inhibitor, and one could hypothesize that disturbing calcium signaling should play a major role in OVCAR3 cell fate. Surprisingly, ABT-737 inhibited ATF4 expression, an observation that has also been made in colorectal (SW480) and ovarian (SKOV3) cell lines [39], and this result warrants further investigation. Nevertheless, even though ATF4 is not overexpressed during combination treatments, its silencing suppressed the expression of Noxa, which protects cells from apoptosis, thus highlighting the crucial role played by the ATF4/Noxa axis in combination-induced cell death. This latter result suggests that this molecular mechanism is involved regardless of the histological subtype of the cell line used.

To evaluate the effectiveness of these innovative combinations in models closer to the clinic, they were tested on tumoroids. PDTO are innovative research models derived from 3-D culture of cancer cells from patient samples that accurately mimic tumor heterogeneity and clinical response to treatments supporting their crucial role in precision medicine [33, 79]. Treatment of two ovarian PDTO lines with thapsigargin/ABT-737 or ONC201/ABT-737 combinations potently triggered apoptosis, highlighting the relevance of these treatments in ovarian cancer management. The difference in sensitivity to treatments observed between the 2 lines might be allocated to the histological background the PDTO come from. Actually, whereas the sensitive line derived from tumor specimen, the more resistant one derived from ascitic cells which are known to grow in a more pro-inflammatory microenvironment conferring them a greater resistance to apoptosis [80].

Partial ovarian PDTO disintegration was accompanied by ER stress/ATF4/Noxa upregulation at transcriptional and translational levels and to our knowledge, it is the first time that this combination was tested and its molecular pathway deciphered in this kind of model. The crucial importance of this transduction pathway in controlling the fate of ovarian PDTO enforces the interest in using these combinations in the clinic.

Taken together, these results identify SERCA2 inhibitors as promising therapeutic options for patients suffering from ovarian cancer and innovative researches are carried on to take up the challenges and to develop SERCA2 inhibitors usable in the clinic [17, 18]. Although a previous work has indicated that ovarian cancer cells do not express PSMA and do not represent favorable candidates to mipsagargin [81], other research avenues such as the development of new SERCA2 inhibitors [82] or the use of the aberrantly expressed ovarian folate receptor as a carrier for thapsigargin analogs (JQ-FT) are underway [16, 83]. ABT-737 oral derivative (ABT-263 – Navitoclax) has passed several phase II clinical trials but its use is limited by an unacceptable thrombocytopenia occurrence [9]. As for ONC201, it is a well‑tolerated, orally administered drug that crosses the blood-brain barrier. It thus combines several advantages that have allowed it to currently be in phase III clinical trials for malignant glioma [27]. Its capacity to synergize with ABT-263 could then relevantly dampen ABT-263 concentrations to avoid adverse thrombocytopenia and the exciting results we obtained with ONC201/ABT-737 combination in PDTO models arouses great opportunity for chemoresistant ovarian cancer management.

## Supplementary information

Supplementary Figure S1

Supplementary Figure S2

Supplementary Figure S3

Supplementary Figure S4

Supplementary Figure S5

Supplementary Figure S6

Original Western Blots

## Data Availability

Supplementary Figs. and the source data underlying the figures are provided as a Supplementary Information file. Other figures or data supporting the results of this study are available from the corresponding author upon reasonable request.

## References

[1] Filho AM, Laversanne M, Ferlay J, Colombet M, Piñeros M, Znaor A, et al. The GLOBOCAN 2022 cancer estimates: data sources, methods, and a snapshot of the cancer burden worldwide. Int J Cancer. 2025;156:1336–46. 39688499 10.1002/ijc.35278

[2] Veneziani AC, Gonzalez-Ochoa E, Alqaisi H, Madariaga A, Bhat G, Rouzbahman M, et al. Heterogeneity and treatment landscape of ovarian carcinoma. Nat Rev Clin Oncol. 2023;20:820–42. 37783747 10.1038/s41571-023-00819-1

[3] Brotin E, Meryet-Figuiere M, Simonin K, Duval RE, Villedieu M, Leroy-Dudal J, et al. Bcl-XL and MCL-1 constitute pertinent targets in ovarian carcinoma and their concomitant inhibition is sufficient to induce apoptosis. Int J Cancer. 2010;126:885–95. 19634140 10.1002/ijc.24787

[4] Del Bufalo D, Damia G. Overview of BH3 mimetics in ovarian cancer. Cancer Treat Rev. 2024;129:102771. 38875743 10.1016/j.ctrv.2024.102771

[5] Vogler M, Braun Y, Smith VM, Westhoff M-A, Pereira RS, Pieper NM, et al. The BCL2 family: from apoptosis mechanisms to new advances in targeted therapy. Signal Transduct Target Ther. 2025;10:91. 40113751 10.1038/s41392-025-02176-0 PMC11926181

[6] van Delft MF, Wei AH, Mason KD, Vandenberg CJ, Chen L, Czabotar PE, et al. The BH3 mimetic ABT-737 targets selective Bcl-2 proteins and efficiently induces apoptosis via Bak/Bax if Mcl-1 is neutralized. Cancer Cell. 2006;10:389–99. 17097561 10.1016/j.ccr.2006.08.027 PMC2953559

[7] Simonin K, Brotin E, Dufort S, Dutoit S, Goux D, N’diaye M, et al. Mcl-1 is an important determinant of the apoptotic response to the BH3-mimetic molecule HA14-1 in cisplatin-resistant ovarian carcinoma cells. Mol Cancer Ther. 2009;8:3162–70. 19887550 10.1158/1535-7163.MCT-09-0493

[8] Simonin K, N’diaye M, Lheureux S, Loussouarn C, Dutoit S, Briand M, et al. Platinum compounds sensitize ovarian carcinoma cells to ABT-737 by modulation of the Mcl-1/Noxa axis. Apoptosis. 2013;18:492–508. 23344663 10.1007/s10495-012-0799-x

[9] Diepstraten ST, Anderson MA, Czabotar PE, Lessene G, Strasser A, Kelly GL. The manipulation of apoptosis for cancer therapy using BH3-mimetic drugs. Nat Rev Cancer. 2022;22:45–64. 34663943 10.1038/s41568-021-00407-4

[10] Petigny-Lechartier C, Duboc C, Jebahi A, Louis MH, Abeilard E, Denoyelle C, et al. The mTORC1/2 inhibitor AZD8055 strengthens the efficiency of the MEK inhibitor trametinib to reduce the Mcl-1/[Bim and Puma] ratio and to sensitize ovarian carcinoma cells to ABT-737. Mol Cancer Ther. 2017;16:102–15. 27980105 10.1158/1535-7163.MCT-16-0342

[11] Monteith GR, Prevarskaya N, Roberts-Thomson SJ. The calcium-cancer signalling nexus. NatRevCancer. 2017;17:367–80. 10.1038/nrc.2017.18 28386091

[12] Prevarskaya N, Skryma R, Shuba Y. Ion channels in cancer: are cancer hallmarks oncochannelopathies? Physiol Rev. 2018;98:559–621. 29412049 10.1152/physrev.00044.2016

[13] Prevarskaya N, Ouadid-Ahidouch H, Skryma R, Shuba Y. Remodelling of Ca2+ transport in cancer: how it contributes to cancer hallmarks?. Philos Trans R Soc Lond B Biol Sci. 2014;369:20130097. 24493745 10.1098/rstb.2013.0097 PMC3917351

[14] Bonnefond M-L, Florent R, Lenoir S, Lambert B, Abeilard E, Giffard F, et al. Inhibition of store-operated channels by carboxyamidotriazole sensitizes ovarian carcinoma cells to anti-BclxL strategies through Mcl-1 down-regulation. Oncotarget. 2018;9:33896–911. 30338034 10.18632/oncotarget.26084 PMC6188062

[15] Bonnefond ML, Lambert B, Giffard F, Abeilard E, Brotin E, Louis MH, et al. Calcium signals inhibition sensitizes ovarian carcinoma cells to anti-Bcl-xL strategies through Mcl-1 down-regulation. Apoptosis. 2015;20:535–50. 25627260 10.1007/s10495-015-1095-3 PMC4348506

[16] Jaskulska A, Janecka AE, Gach-Janczak K. Thapsigargin-from traditional medicine to anticancer drug. Int J Mol Sci. 2020;22:4. 33374919 10.3390/ijms22010004 PMC7792614

[17] Isaacs JT, Brennen WN, Christensen SB, Denmeade SR. Mipsagargin: the beginning-not the end-of thapsigargin prodrug-based cancer therapeutics. Molecules. 2021;26:7469. 34946547 10.3390/molecules26247469 PMC8707208

[18] Chemaly ER, Troncone L, Lebeche D. SERCA control of cell death and survival. Cell Calcium. 2018;69:46–61. 28747251 10.1016/j.ceca.2017.07.001 PMC5748262

[19] Pakos-Zebrucka K, Koryga I, Mnich K, Ljujic M, Samali A, Gorman AM. The integrated stress response. EMBO Rep. 2016;17:1374–95. 27629041 10.15252/embr.201642195 PMC5048378

[20] Pihán P, Carreras-Sureda A, Hetz C. BCL-2 family: integrating stress responses at the ER to control cell demise. Cell Death Differ. 2017;24:1478–87. 28622296 10.1038/cdd.2017.82 PMC5563989

[21] Ma Z, Fan C, Yang Y, Di S, Hu W, Li T, et al. Thapsigargin sensitizes human esophageal cancer to TRAIL-induced apoptosis via AMPK activation. Sci Rep. 2016;6:35196. 27731378 10.1038/srep35196 PMC5059685

[22] Wang L, Wang L, Song R, Shen Y, Sun Y, Gu Y, et al. Targeting sarcoplasmic/endoplasmic reticulum Ca^2^+-ATPase 2 by curcumin induces ER stress-associated apoptosis for treating human liposarcoma. Mol Cancer Ther. 2011;10:461–71. 21282356 10.1158/1535-7163.MCT-10-0812

[23] Li W, Ouyang Z, Zhang Q, Wang L, Shen Y, Wu X, et al. SBF-1 exerts strong anticervical cancer effect through inducing endoplasmic reticulum stress-associated cell death via targeting sarco/endoplasmic reticulum Ca(2+)-ATPase 2. Cell Death Dis. 2014;5:e1581. 25522275 10.1038/cddis.2014.538 PMC4649847

[24] Florent R, Weiswald L-B, Lambert B, Brotin E, Abeilard E, Louis M-H, et al. Bim, Puma and Noxa upregulation by Naftopidil sensitizes ovarian cancer to the BH3-mimetic ABT-737 and the MEK inhibitor Trametinib. Cell Death Dis. 2020;11:380. 32424251 10.1038/s41419-020-2588-8 PMC7235085

[25] McLean K, VanDeVen NA, Sorenson DR, Daudi S, Liu JR. The HIV protease inhibitor saquinavir induces endoplasmic reticulum stress, autophagy, and apoptosis in ovarian cancer cells. Gynecol Oncol. 2009;112:623–30. 19147209 10.1016/j.ygyno.2008.11.028

[26] Yoo JY, Hurwitz BS, Bolyard C, Yu J-G, Zhang J, Selvendiran K, et al. Bortezomib-induced unfolded protein response increases oncolytic HSV-1 replication resulting in synergistic antitumor effects. Clin Cancer Res J Am Assoc Cancer Res. 2014;20:3787–98. 10.1158/1078-0432.CCR-14-0553 PMC4132885 24815720

[27] Prabhu VV, Morrow S, Rahman Kawakibi A, Zhou L, Ralff M, Ray J, et al. ONC201 and imipridones: anti-cancer compounds with clinical efficacy. Neoplasia. 2020;22:725–44. 33142238 10.1016/j.neo.2020.09.005 PMC7588802

[28] Fan Y, Wang J, Fang Z, Pierce SR, West L, Staley A, et al. Anti-tumor and anti-invasive effects of ONC201 on ovarian cancer cells and a transgenic mouse model of serous ovarian cancer. Front Oncol. 2022;12:789450. 35372029 10.3389/fonc.2022.789450 PMC8970020

[29] Rumman M, Buck S, Polin L, Dzinic S, Boerner J, Winer IS. ONC201 induces the unfolded protein response (UPR) in high- and low-grade ovarian carcinoma cell lines and leads to cell death regardless of platinum sensitivity. Cancer Med. 2021;10:3373–87. 33932119 10.1002/cam4.3858 PMC8124100

[30] Arrillaga-Romany I, Lassman A, McGovern SL, Mueller S, Nabors B, van den Bent M, et al. ACTION: a randomized phase 3 study of ONC201 (dordaviprone) in patients with newly diagnosed H3 K27M-mutant diffuse glioma. Neuro-Oncol. 2024;26:S173–81. 38445964 10.1093/neuonc/noae031 PMC11066938

[31] Barnes BM, Nelson L, Tighe A, Burghel GJ, Lin I-H, Desai S, et al. Distinct transcriptional programs stratify ovarian cancer cell lines into the five major histological subtypes. Genome Med. 2021;13:140. 34470661 10.1186/s13073-021-00952-5 PMC8408985

[32] Poulain L, Lincet H, Duigou F, Deslandes E, Sichel F, Gauduchon P, et al. Acquisition of chemoresistance in a human ovarian carcinoma cell is linked to a defect in cell cycle control. Int J Cancer. 1998;78:454–63. 9797134 10.1002/(sici)1097-0215(19981109)78:4<454::aid-ijc11>3.0.co;2-6

[33] Thorel L, Dolivet E, Morice PM, Florent R, Divoux J, Perréard M, et al. Long-term patient-derived ovarian cancer organoids closely recapitulate tumor of origin and clinical response. J Exp Clin Cancer Res. 2025;44:282. 41057919 10.1186/s13046-025-03537-x PMC12502492

[34] Thorel L, Divoux J, Lequesne J, Babin G, Morice P-M, Florent R, et al. The OVAREX study: establishment of ex vivo ovarian cancer models to validate innovative therapies and to identify predictive biomarkers. BMC Cancer. 2024;24:701. 38849726 10.1186/s12885-024-12429-w PMC11157894

[35] Mahn K, Hirst SJ, Ying S, Holt MR, Lavender P, Ojo OO, et al. Diminished sarco/endoplasmic reticulum Ca2+ ATPase (SERCA) expression contributes to airway remodelling in bronchial asthma. Proc Natl Acad Sci USA. 2009;106:10775–80. 19541629 10.1073/pnas.0902295106 PMC2699374

[36] Iurlaro R, Muñoz-Pinedo C. Cell death induced by endoplasmic reticulum stress. FEBS J. 2016;283:2640–52. 26587781 10.1111/febs.13598

[37] Wortel IMN, van der Meer LT, Kilberg MS, van Leeuwen FN. Surviving stress: modulation of ATF4-mediated stress responses in normal and malignant cells. Trends Endocrinol Metab TEM. 2017;28:794–806. 28797581 10.1016/j.tem.2017.07.003 PMC5951684

[38] Domcke S, Sinha R, Levine DA, Sander C, Schultz N. Evaluating cell lines as tumour models by comparison of genomic profiles. Nat Commun. 2013;4:2126. 23839242 10.1038/ncomms3126 PMC3715866

[39] Di Cristofano FR, Fong MW, Huntington KE, Carneiro BA, Zhou L, El-Deiry WS. Synergistic activity of ABT-263 and ONC201/TIC10 against solid tumor cell lines is associated with suppression of anti-apoptotic Mcl-1, BAG3, pAkt, and upregulation of pro-apoptotic Noxa and Bax cleavage during apoptosis. Am J Cancer Res. 2023;13:307–25. 36777502 PMC9906082

[40] Bertocci LA, Rovatti JR, Wu A, Morey A, Bose DD, Kinney SRM. Calcium handling genes are regulated by promoter DNA methylation in colorectal cancer cells. Eur J Pharm. 2022;915:174698. 10.1016/j.ejphar.2021.174698 34896109

[41] Arbabian A, Brouland J-P, Apáti Á, Pászty K, Hegedűs L, Enyedi Á, et al. Modulation of endoplasmic reticulum calcium pump expression during lung cancer cell differentiation. FEBS J. 2013;280:5408–18. 23157274 10.1111/febs.12064

[42] Li J, Li X, Huang H, Tao L, Zhang C, Xie Y, et al. Role of SERCA3 in the prognosis and immune function in pan-cancer. J Oncol. 2022;2022:9359879. 36385955 10.1155/2022/9359879 PMC9652089

[43] Einbond LS, Wu H, Sandu C, Ford M, Mighty J, Antonetti V, et al. Digitoxin enhances the growth inhibitory effects of thapsigargin and simvastatin on ER negative human breast cancer cells. Fitoterapia. 2016;109:146–54. 26691294 10.1016/j.fitote.2015.12.005

[44] Bellizzi Y, Cornier PG, Delpiccolo CML, Mata EG, Blank V, Roguin LP. Synergistic antitumor effect of a penicillin derivative combined with thapsigargin in melanoma cells. J Cancer Res Clin Oncol. 2022;148:3361–73. 35751681 10.1007/s00432-022-04129-4 PMC11801134

[45] Chen Y-C, Yang C-W, Chan T-F, Farooqi AA, Chang H-S, Yen C-H, et al. Cryptocaryone promotes ROS-dependent antiproliferation and apoptosis in ovarian cancer cells. Cells. 2022;11:641. 35203294 10.3390/cells11040641 PMC8870566

[46] Gloaguen, Voisin-Chiret C, Sopkova-de AS, Oliveira SJ, Fogha J, Gautier F, et al. First evidence that oligopyridines, alpha-helix foldamers, inhibit Mcl-1 and sensitize ovarian carcinoma cells to Bcl-xL-targeting strategies. J Med Chem. 2015;58:1644–68. 25585174 10.1021/jm500672y

[47] Wu L, Huang X, Kuang Y, Xing Z, Deng X, Luo Z. Thapsigargin induces apoptosis in adrenocortical carcinoma by activating endoplasmic reticulum stress and the JNK signaling pathway: an in vitro and in vivo study. Drug Des Dev Ther. 2019;13:2787–98. 10.2147/DDDT.S209947 PMC6697672 31496655

[48] Sehgal P, Szalai P, Olesen C, Praetorius HA, Nissen P, Christensen SB, et al. Inhibition of the sarco/endoplasmic reticulum (ER) Ca2+-ATPase by thapsigargin analogs induces cell death via ER Ca2+ depletion and the unfolded protein response. J Biol Chem. 2017;292:19656–73. 28972171 10.1074/jbc.M117.796920 PMC5712609

[49] Wang F, Liu D, Xu H, Li Y, Wang W, Liu B, et al. Thapsigargin induces apoptosis by impairing cytoskeleton dynamics in human lung adenocarcinoma cells. ScientificWorldJournal. 2014;2014:619050. 24605059 10.1155/2014/619050 PMC3926280

[50] Lindner P, Christensen SB, Nissen P, Møller JV, Engedal N. Cell death induced by the ER stressor thapsigargin involves death receptor 5, a non-autophagic function of MAP1LC3B, and distinct contributions from unfolded protein response components. Cell Commun Signal CCS. 2020;18:12. 31987044 10.1186/s12964-019-0499-z PMC6986015

[51] Puthalakath H, O’Reilly LA, Gunn P, Lee L, Kelly PN, Huntington ND, et al. ER stress triggers apoptosis by activating BH3-only protein Bim. Cell. 2007;129:1337–49. 17604722 10.1016/j.cell.2007.04.027

[52] Zhang L, Lopez H, George NM, Liu X, Pang X, Luo X. Selective involvement of BH3-only proteins and differential targets of Noxa in diverse apoptotic pathways. Cell Death Differ. 2011;18:864–73. 21113147 10.1038/cdd.2010.152 PMC3074052

[53] Cano-González A, Mauro-Lizcano M, Iglesias-Serret D, Gil J, López-Rivas A. Involvement of both caspase-8 and Noxa-activated pathways in endoplasmic reticulum stress-induced apoptosis in triple-negative breast tumor cells. Cell Death Dis. 2018;9:134. 29374147 10.1038/s41419-017-0164-7 PMC5833688

[54] Gomez-Bougie P, Halliez M, Moreau P, Pellat-Deceunynck C, Amiot M. Repression of Mcl-1 and disruption of the Mcl-1/Bak interaction in myeloma cells couple ER stress to mitochondrial apoptosis. Cancer Lett. 2016;383:204–11. 27697610 10.1016/j.canlet.2016.09.030

[55] Li J, Lee B, Lee AS. Endoplasmic reticulum stress-induced apoptosis: multiple pathways and activation of p53-up-regulated modulator of apoptosis (PUMA) and NOXA by p53. J Biol Chem. 2006;281:7260–70. 16407291 10.1074/jbc.M509868200

[56] Soderquist R, Pletnev AA, Danilov AV, Eastman A. The putative BH3 mimetic S1 sensitizes leukemia to ABT-737 by increasing reactive oxygen species, inducing endoplasmic reticulum stress, and upregulating the BH3-only protein NOXA. Apoptosis. 2014;19:201–9. 24072590 10.1007/s10495-013-0910-y PMC3947354

[57] Sakthivel D, Bolívar BE, Bouchier-Hayes L. Cellular autophagy, an unbidden effect of caspase inhibition by zVAD-fmk. FEBS J. 2022;289:3097–100. 35043564 10.1111/febs.16346 PMC9177521

[58] Kishino A, Hayashi K, Maeda M, Jike T, Hidai C, Nomura Y, et al. Caspase-8 regulates endoplasmic reticulum stress-induced necroptosis independent of the apoptosis pathway in auditory cells. Int J Mol Sci. 2019;20:5896. 31771290 10.3390/ijms20235896 PMC6928907

[59] Wang H, Yang Y-B, Shen H-M, Gu J, Li T, Li X-M. ABT-737 induces Bim expression via JNK signaling pathway and its effect on the radiation sensitivity of HeLa cells. PLoS ONE. 2012;7:e52483. 23285061 10.1371/journal.pone.0052483 PMC3527555

[60] Le PJ, Maillet L, Sarosiek K, Vuillier C, Gautier F, Montessuit S, et al. Constitutive p53 heightens mitochondrial apoptotic priming and favors cell death induction by BH3 mimetic inhibitors of BCL-xL. Cell Death Dis. 2016;7:e2083. 26844698 10.1038/cddis.2015.400 PMC4849148

[61] Morales AA, Kurtoglu M, Matulis SM, Liu J, Siefker D, Gutman DM, et al. Distribution of Bim determines Mcl-1 dependence or codependence with Bcl-xL/Bcl-2 in Mcl-1-expressing myeloma cells. Blood. 2011;118:1329–39. 21659544 10.1182/blood-2011-01-327197 PMC3152498

[62] Geserick P, Wang J, Feoktistova M, Leverkus M. The ratio of Mcl-1 and Noxa determines ABT737 resistance in squamous cell carcinoma of the skin. Cell Death Dis. 2014;5:e1412. 25210795 10.1038/cddis.2014.379 PMC4540197

[63] Tromp JM, Geest CR, Breij EC, Elias JA, van LJ, Luijks DM, et al. Tipping the Noxa/Mcl-1 balance overcomes ABT-737 resistance in chronic lymphocytic leukemia. Clin Cancer Res. 2012;18:487–98. 22128299 10.1158/1078-0432.CCR-11-1440

[64] Núñez-Vázquez S, Sánchez-Vera I, Saura-Esteller J, Cosialls AM, Noisier AFM, Albericio F, et al. NOXA upregulation by the prohibitin-binding compound fluorizoline is transcriptionally regulated by integrated stress response-induced ATF3 and ATF4. FEBS J. 2021;288:1271–85. 32648994 10.1111/febs.15480

[65] Sharma K, Vu T-T, Cook W, Naseri M, Zhan K, Nakajima W, et al. p53-independent Noxa induction by cisplatin is regulated by ATF3/ATF4 in head and neck squamous cell carcinoma cells. Mol Oncol. 2018;12:788–98. 29352505 10.1002/1878-0261.12172 PMC5983129

[66] Yan J, Zhong N, Liu G, Chen K, Liu X, Su L, et al. Usp9x- and Noxa-mediated Mcl-1 downregulation contributes to pemetrexed-induced apoptosis in human non-small-cell lung cancer cells. Cell Death Dis. 2014;5:e1316. 24991768 10.1038/cddis.2014.281 PMC4123075

[67] Gadet R, Jabbour L, Nguyen TTM, Lohez O, Mikaelian I, Gonzalo P, et al. The endoplasmic reticulum pool of Bcl-xL prevents cell death through IP3R-dependent calcium release. Cell Death Discov. 2024;10:1–11. 39090104 10.1038/s41420-024-02112-1 PMC11294475

[68] Rosa N, Ivanova H, Wagner LE, Kale J, La Rovere R, Welkenhuyzen K, et al. Bcl-xL acts as an inhibitor of IP3R channels, thereby antagonizing Ca2+-driven apoptosis. Cell Death Differ. 2022;29:788–805. 34750538 10.1038/s41418-021-00894-w PMC8990011

[69] Yang J, Vais H, Gu W, Foskett JK. Biphasic regulation of InsP3 receptor gating by dual Ca2+ release channel BH3-like domains mediates Bcl-xL control of cell viability. Proc Natl Acad Sci USA. 2016;113:E1953–1962. 26976600 10.1073/pnas.1517935113 PMC4822637

[70] Albershardt TC, Salerni BL, Soderquist RS, Bates DJ, Pletnev AA, Kisselev AF, et al. Multiple BH3 mimetics antagonize antiapoptotic MCL1 protein by inducing the endoplasmic reticulum stress response and up-regulating BH3-only protein NOXA. J Biol Chem. 2011;286:24882–95. 21628457 10.1074/jbc.M111.255828 PMC3137063

[71] Zhang B, Yan Y, Li Y, Zhang D, Zeng J, Wang L, et al. Dimethyl celecoxib sensitizes gastric cancer cells to ABT-737 via AIF nuclear translocation. J Cell Mol Med. 2016;20:2148–59. 27374973 10.1111/jcmm.12913 PMC5082400

[72] Antignani A, Sarnovsky R, FitzGerald DJ. ABT-737 promotes the dislocation of ER luminal proteins to the cytosol, including pseudomonas exotoxin. Mol Cancer Ther. 2014;13:1655–63. 24739394 10.1158/1535-7163.MCT-13-0998 PMC6258188

[73] Yan T, Ma X, Guo L, Lu R. Targeting endoplasmic reticulum stress signaling in ovarian cancer therapy. Cancer Biol Med. 2023;20:748–64. 37817482 10.20892/j.issn.2095-3941.2023.0232 PMC10618951

[74] Kline CLB, Van den Heuvel APJ, Allen JE, Prabhu VV, Dicker DT, El-Deiry WS. ONC201 kills solid tumor cells by triggering an integrated stress response dependent on ATF4 activation by specific eIF2α kinases. Sci Signal. 2016;9:ra18. 26884600 10.1126/scisignal.aac4374 PMC4968406

[75] Tu Y-S, He J, Liu H, Lee HC, Wang H, Ishizawa J, et al. The imipridone ONC201 induces apoptosis and overcomes chemotherapy resistance by up-regulation of bim in multiple myeloma. Neoplasia. 2017;19:772–80. 28863346 10.1016/j.neo.2017.07.009 PMC5577403

[76] Fang Z, Wang J, Clark LH, Sun W, Yin Y, Kong W, et al. ONC201 demonstrates anti-tumorigenic and anti-metastatic activity in uterine serous carcinoma in vitro. Am J Cancer Res. 2018;8:1551–63. 30210923 PMC6129479

[77] Ishizawa J, Kojima K, Chachad D, Ruvolo P, Ruvolo V, Jacamo RO, et al. ATF4 induction through an atypical integrated stress response to ONC201 triggers p53-independent apoptosis in hematological malignancies. Sci Signal. 2016;9:ra17. 26884599 10.1126/scisignal.aac4380 PMC4815038

[78] Karpel-Massler G, Bâ M, Shu C, Halatsch M-E, Westhoff M-A, Bruce JN, et al. TIC10/ONC201 synergizes with Bcl-2/Bcl-xL inhibition in glioblastoma by suppression of Mcl-1 and its binding partners in vitro and in vivo. Oncotarget. 2015;6:36456–71. 26474387 10.18632/oncotarget.5505 PMC4742189

[79] Thorel L, Perréard M, Florent R, Divoux J, Coffy S, Vincent A, et al. Patient-derived tumor organoids: a new avenue for preclinical research and precision medicine in oncology. Exp Mol Med. 2024;56:1531–51. 38945959 10.1038/s12276-024-01272-5 PMC11297165

[80] Carmi YK, Agbarya A, Khamaisi H, Farah R, Shechtman Y, Korobochka R, et al. Ovarian cancer ascites confers platinum chemoresistance to ovarian cancer cells. Transl Oncol. 2024;44:101939. 38489872 10.1016/j.tranon.2024.101939 PMC10955424

[81] Aide N, Poulain L, Elie N, Briand M, Giffard F, Blanc-Fournier C, et al. A PSMA-targeted theranostic approach is unlikely to be efficient in serous ovarian cancers. EJNMMI Res. 2021;11:11. 33559764 10.1186/s13550-021-00756-z PMC7873152

[82] Marchesini M, Gherli A, Montanaro A, Patrizi L, Sorrentino C, Pagliaro L, et al. Blockade of oncogenic NOTCH1 with the SERCA inhibitor CAD204520 in T cell acute lymphoblastic leukemia. Cell Chem Biol. 2020;27:678–6.e13. 32386594 10.1016/j.chembiol.2020.04.002 PMC7305996

[83] Roti G, Qi J, Kitara S, Sanchez-Martin M, Saur Conway A, Varca AC, et al. Leukemia-specific delivery of mutant NOTCH1 targeted therapy. J Exp Med. 2018;215:197–21. 29158376 10.1084/jem.20151778 PMC5748843

