# Supplementary figures and images for: UPR/ATF4/Noxa pathway overactivation through SERCA2 inhibition or ONC201 treatment combined with ABT-737 triggers apoptosis in chemoresistant ovarian cancer cells and patient-derived tumor organoids
Source: Cell Death Dis. 2026 Mar 27;17(1):416. doi: 10.1038/s41419-026-08559-7 (PMC13149518; doi:10.1038/s41419-026-08559-7)

**A**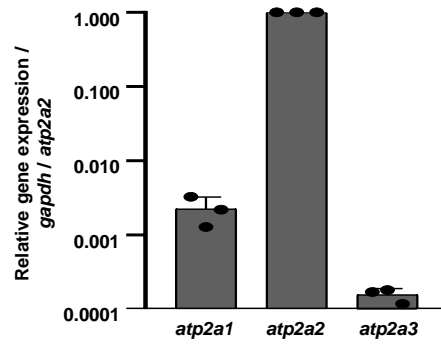**B**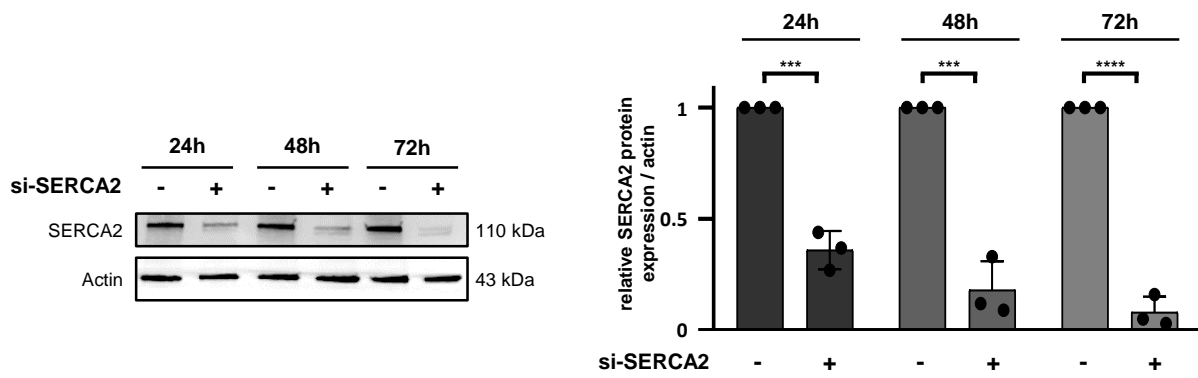**C**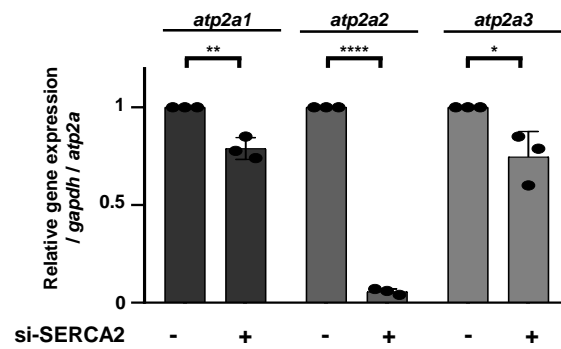

Supplement: Supplementary file 1 — Supplementary Figure S1 [file 41419_2026_8559_MOESM1_ESM.pdf]

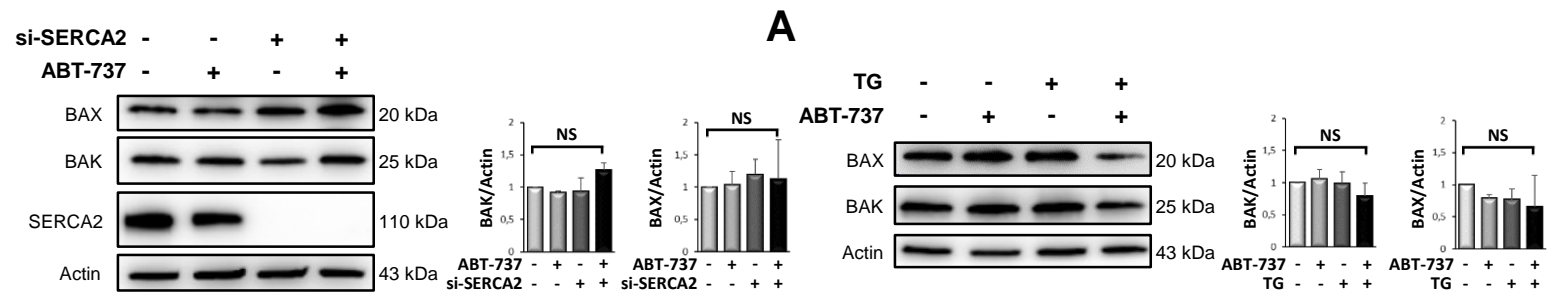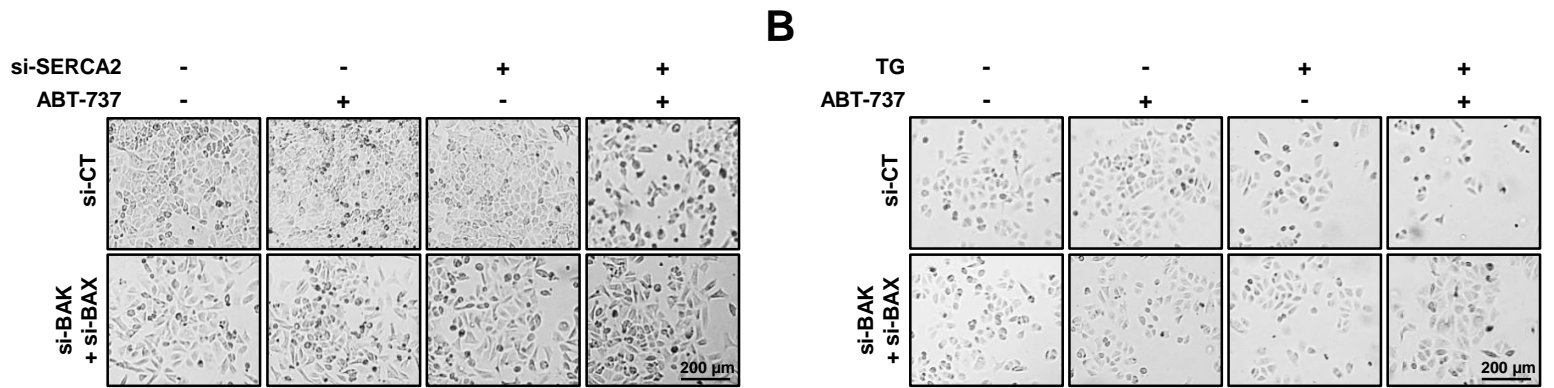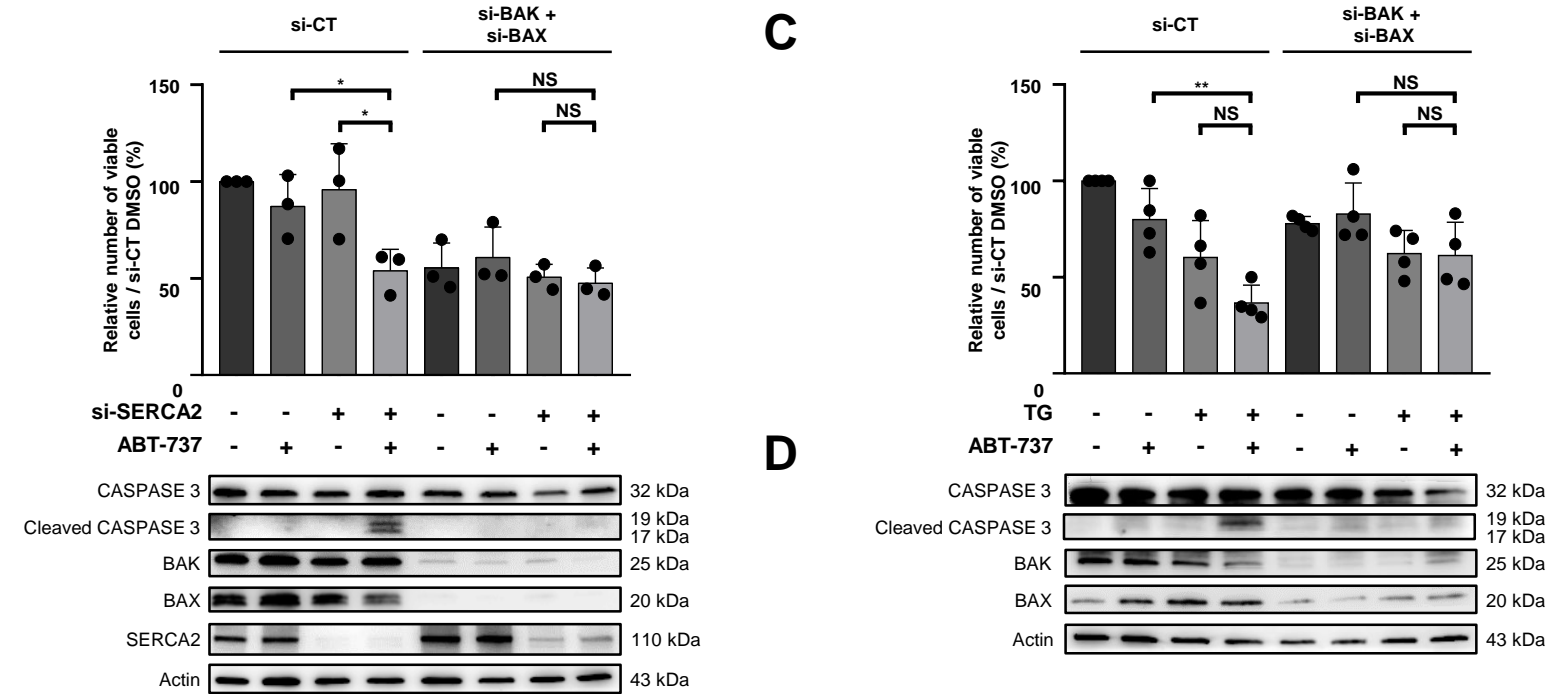

Supplement: Supplementary file 2 — Supplementary Figure S2 [file 41419_2026_8559_MOESM2_ESM.pdf]

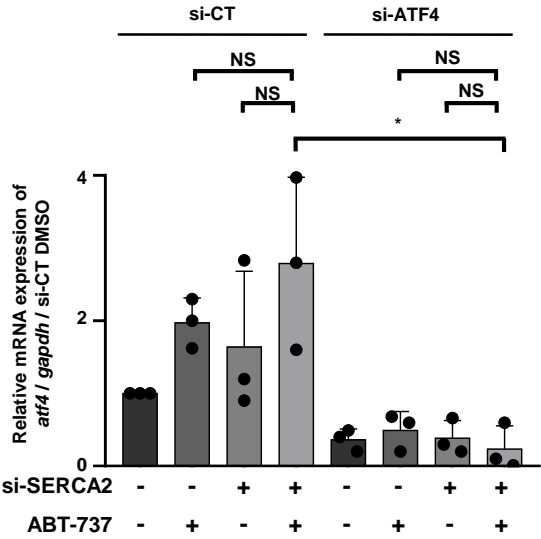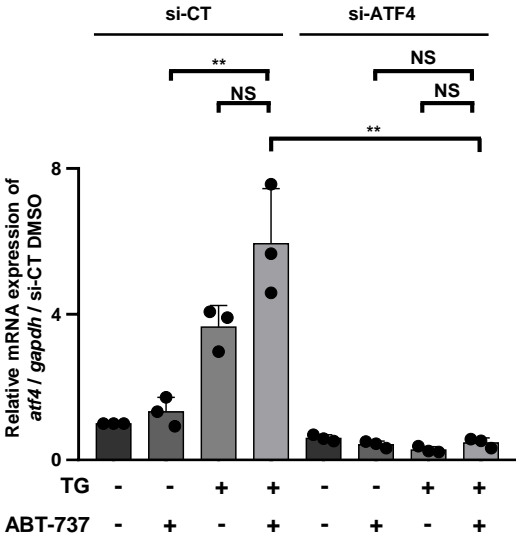

Supplement: Supplementary file 3 — Supplementary Figure S3 [file 41419_2026_8559_MOESM3_ESM.pdf]

**A**

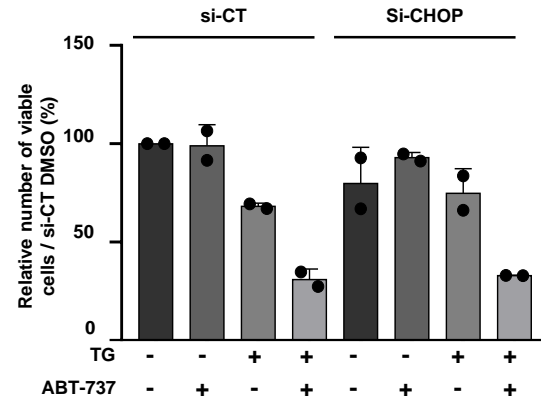

# B

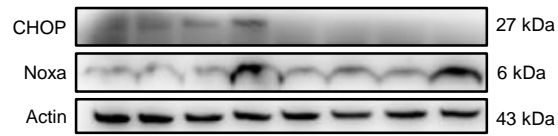

Supplement: Supplementary file 4 — Supplementary Figure S4 [file 41419_2026_8559_MOESM4_ESM.pdf]

**A**

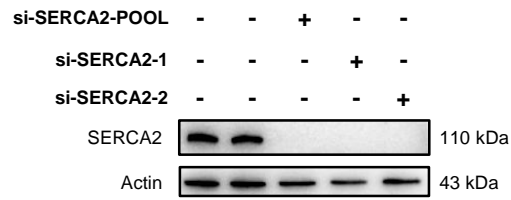

**B**

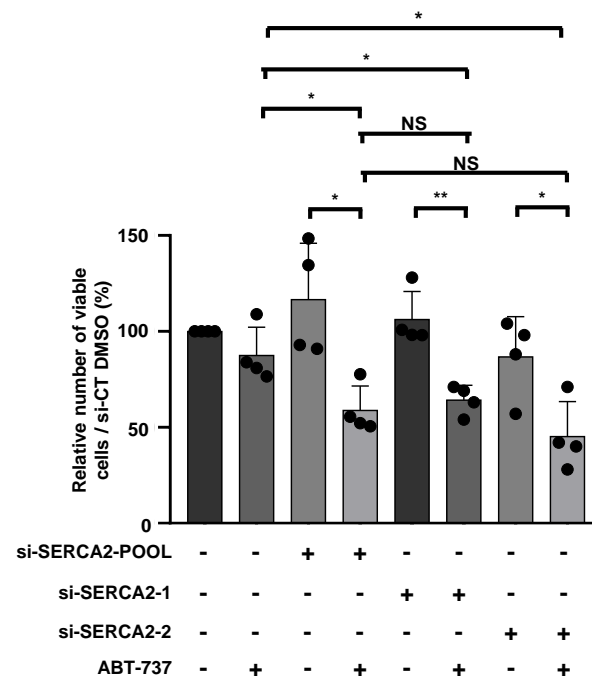

**C**

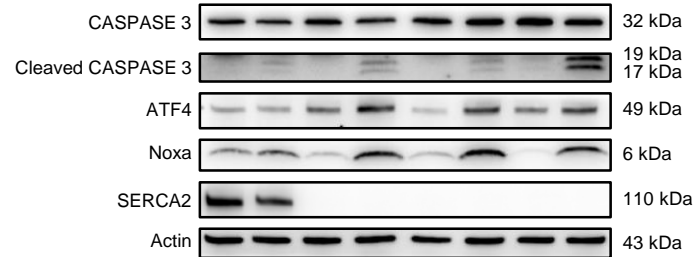

Supplement: Supplementary file 5 — Supplementary Figure S5 [file 41419_2026_8559_MOESM5_ESM.pdf]

# OVCAR3

**A**

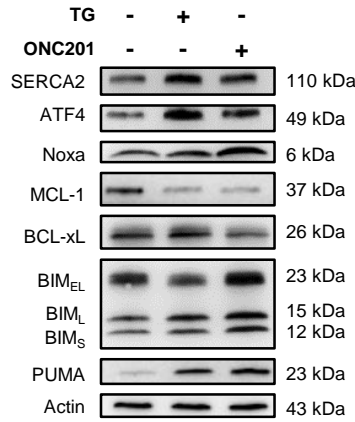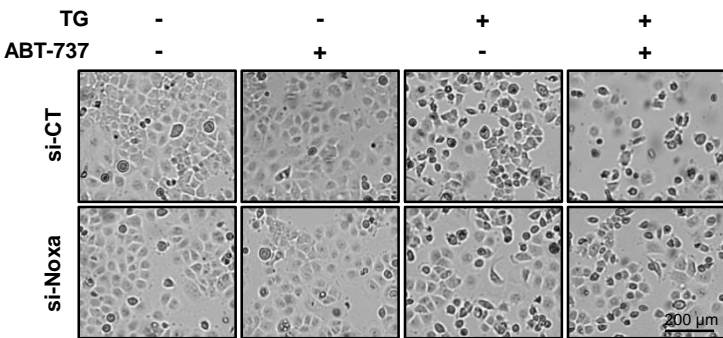

**B**

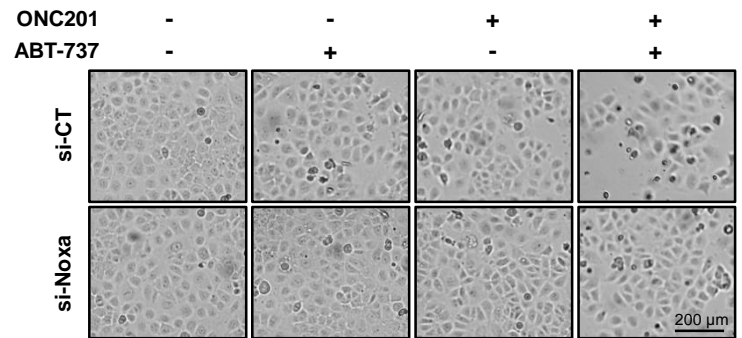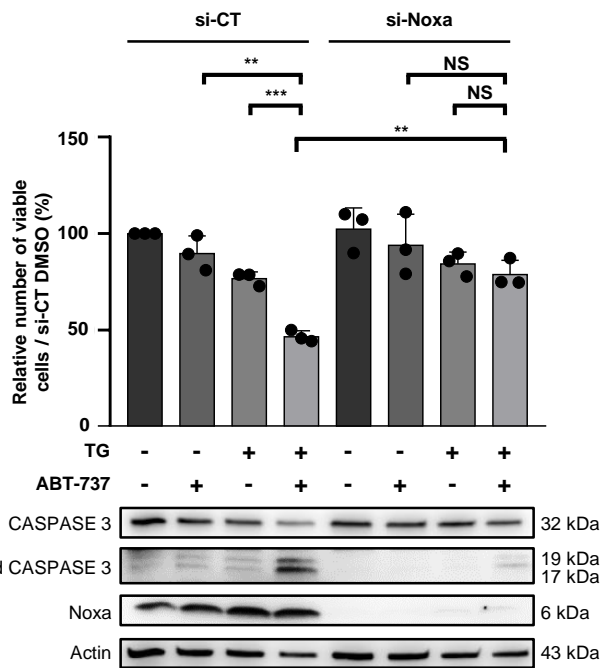

**C**

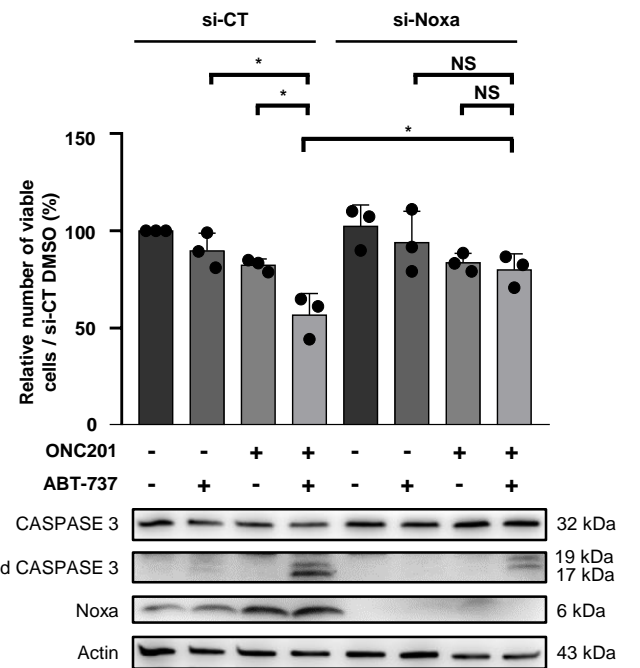

**D**

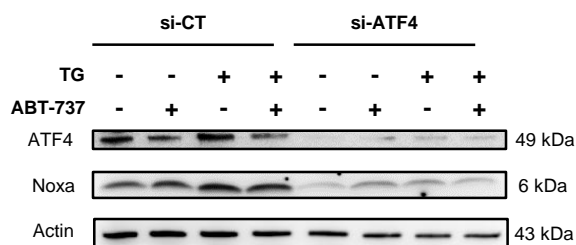

**E**

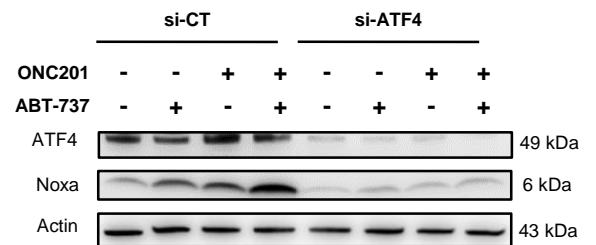

Supplement: Supplementary file 6 — Supplementary Figure S6 [file 41419_2026_8559_MOESM6_ESM.pdf]
