# Supplementary material for: UPR/ATF4/Noxa pathway overactivation through SERCA2 inhibition or ONC201 treatment combined with ABT-737 triggers apoptosis in chemoresistant ovarian cancer cells and patient-derived tumor organoids
Source: Cell Death Dis. 2026 Mar 27;17(1):416. doi: 10.1038/s41419-026-08559-7 (PMC13149518; doi:10.1038/s41419-026-08559-7)

Figures 1C

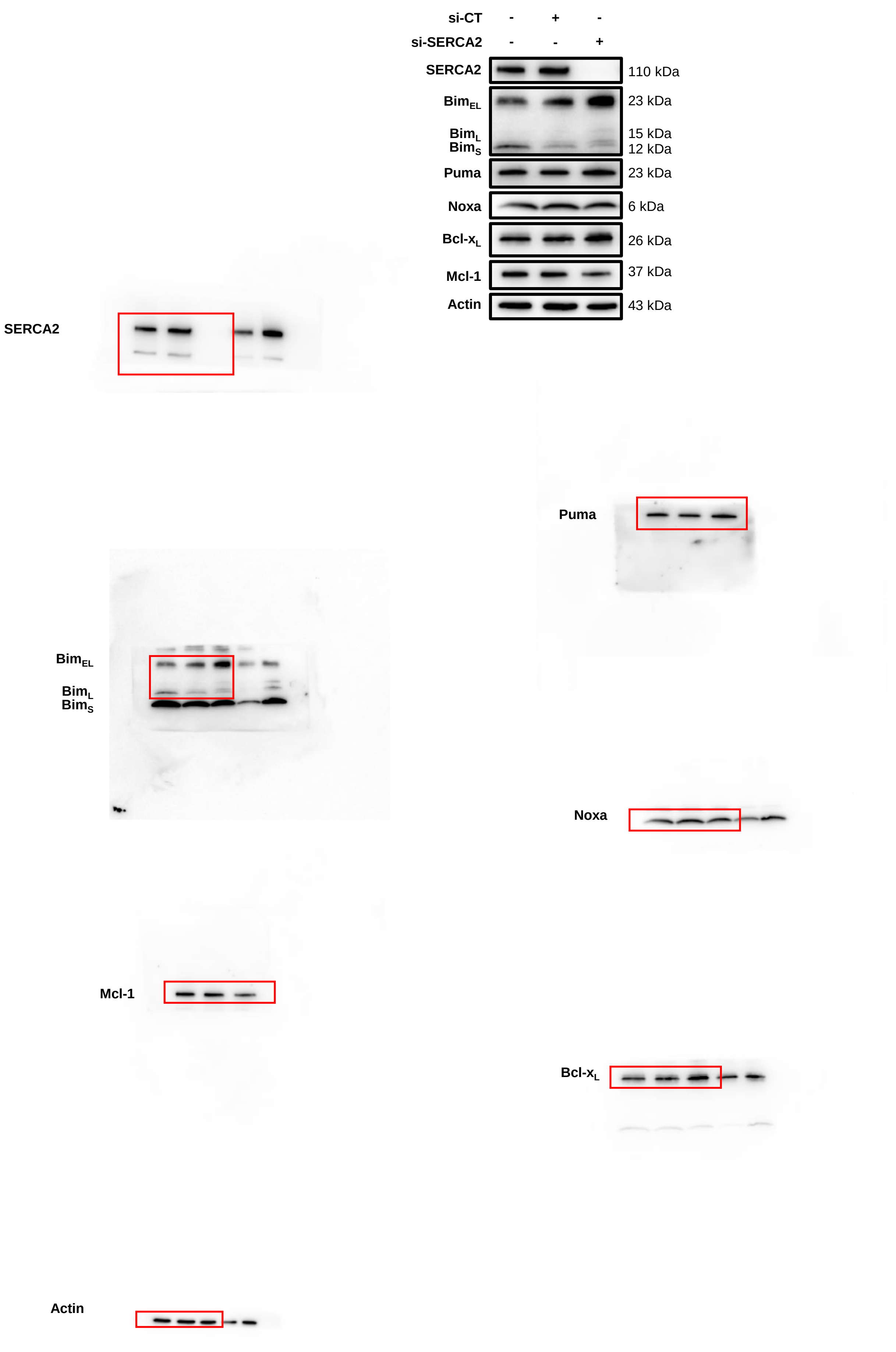
si-CT - + -
si-SERCA2 - - +
SERCA2
110 kDa
Bim$_{EL}$
23 kDa
Bim$_{L}$
15 kDa
Bim$_{S}$
12 kDa
Puma
23 kDa
Noxa
6 kDa
Bcl-x$_{L}$
26 kDa
Mcl-1
37 kDa
Actin
43 kDa
SERCA2
Puma
Bim$_{EL}$
Bim$_{L}$
Bim$_{S}$
Noxa
Mcl-1
Bcl-x$_{L}$
Actin

Figures 1C

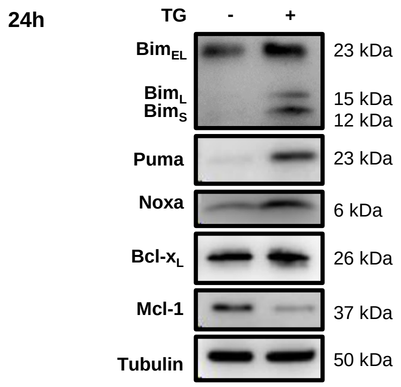
24h
TG
-
+
Bim_{EL}
Bim_{L}
Bim_{S}
Puma
Noxa
Bcl-x_L
Mcl-1
Tubulin
23 kDa
15 kDa
12 kDa
23 kDa
6 kDa
26 kDa
37 kDa
50 kDa

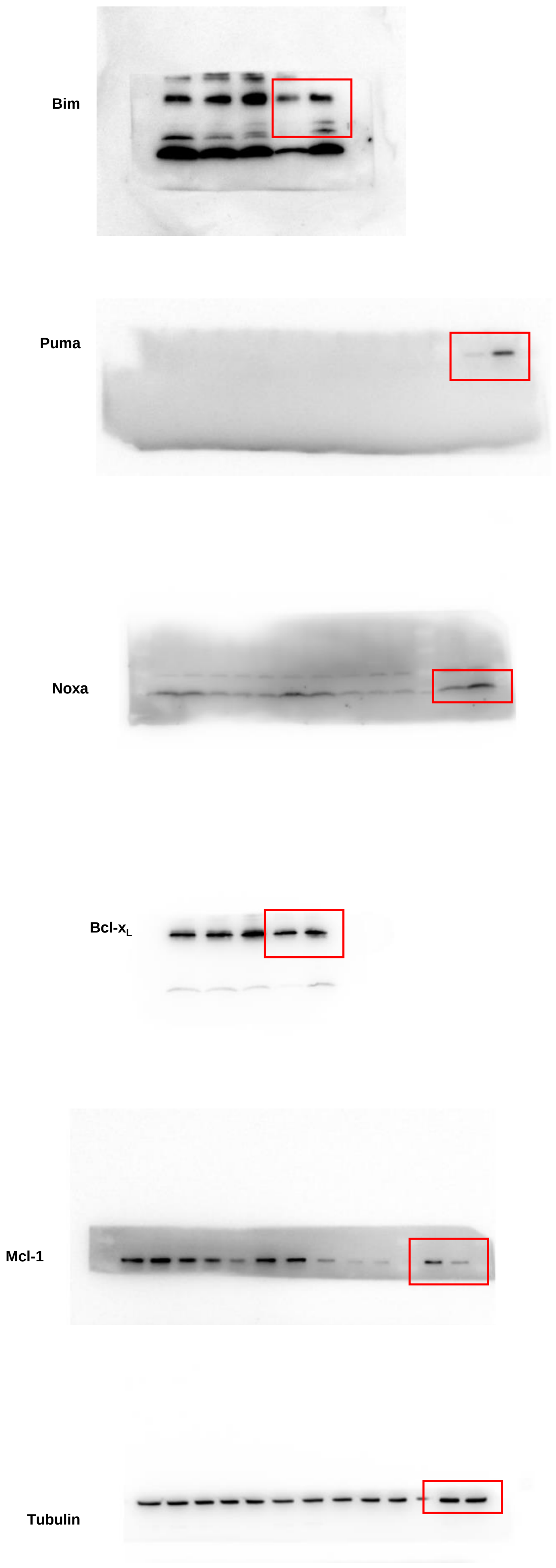
Bim
Puma
Noxa
Bcl-x_L
Mcl-1
Tubulin

Figures 2B

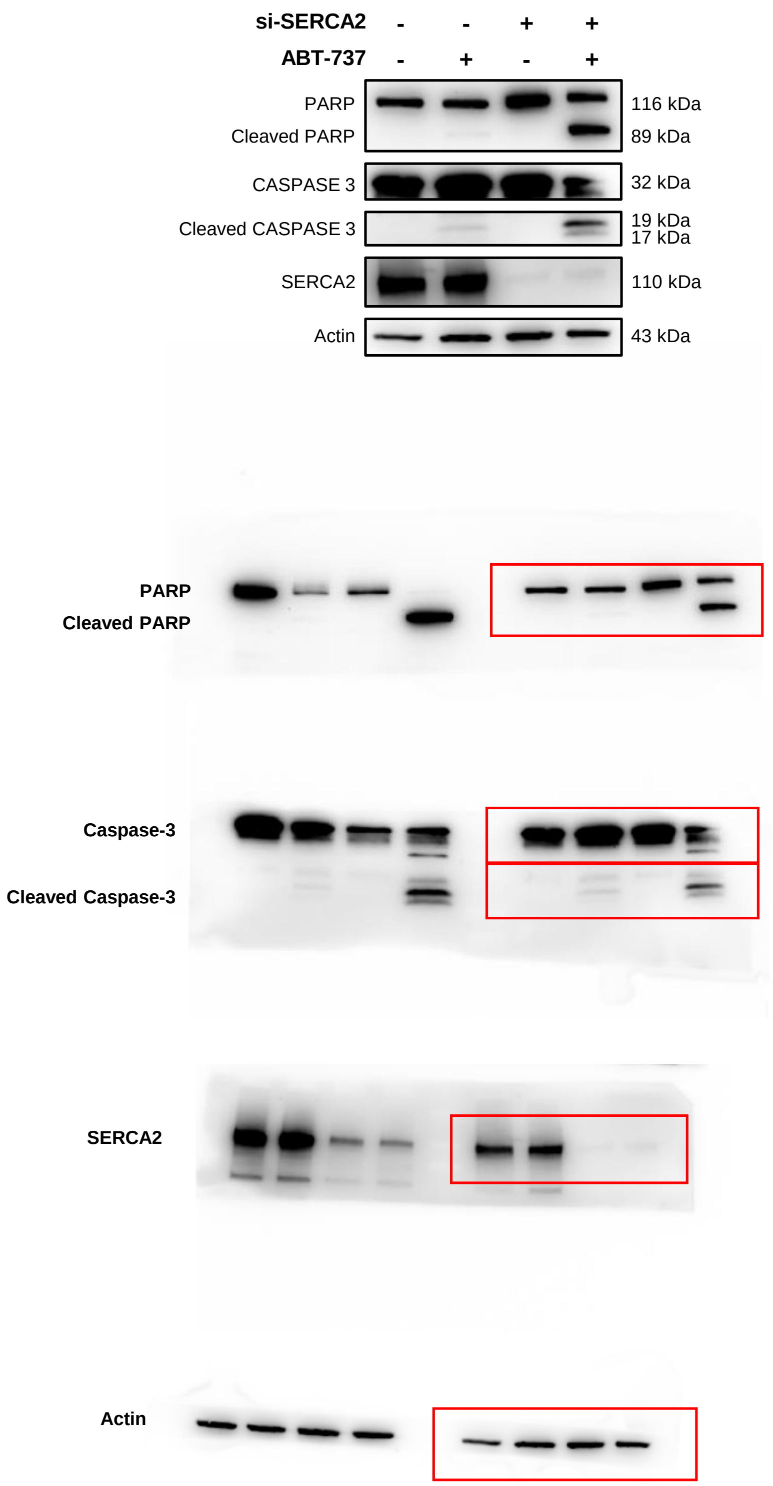

si-SERCA2 - - + +
ABT-737 - + - +
PARP
Cleaved PARP
116 kDa
89 kDa
CASPASE 3
32 kDa
Cleaved CASPASE 3
19 kDa
17 kDa
SERCA2
110 kDa
Actin
43 kDa
PARP
Cleaved PARP
Caspase-3
Cleaved Caspase-3
SERCA2
Actin

Figures 2B

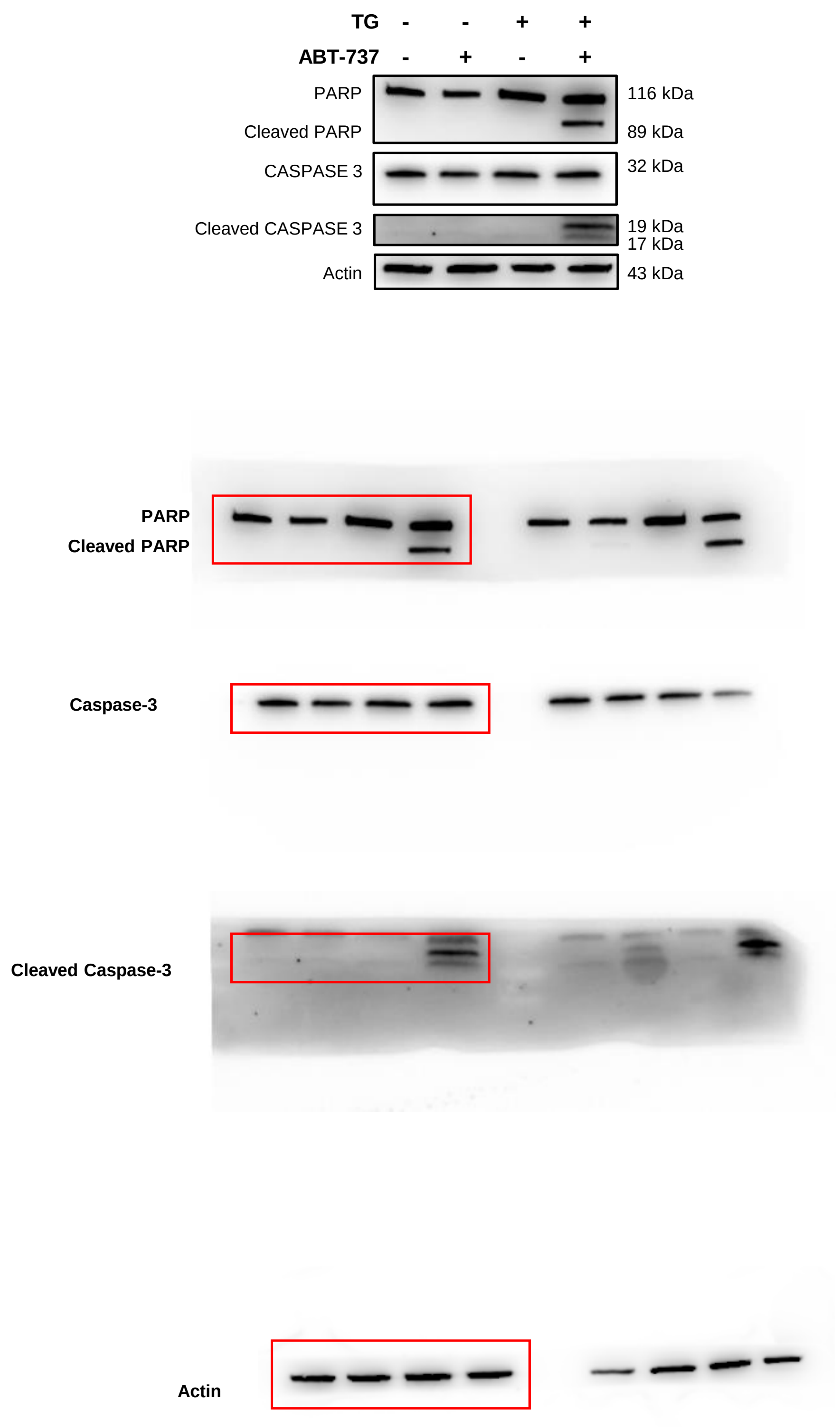
TG - - + +
ABT-737 - + - +
PARP
Cleaved PARP
CASPASE 3
Cleaved CASPASE 3
Actin
116 kDa
89 kDa
32 kDa
19 kDa
17 kDa
43 kDa
PARP
Cleaved PARP
Caspase-3
Cleaved Caspase-3
Actin

Figures 2E

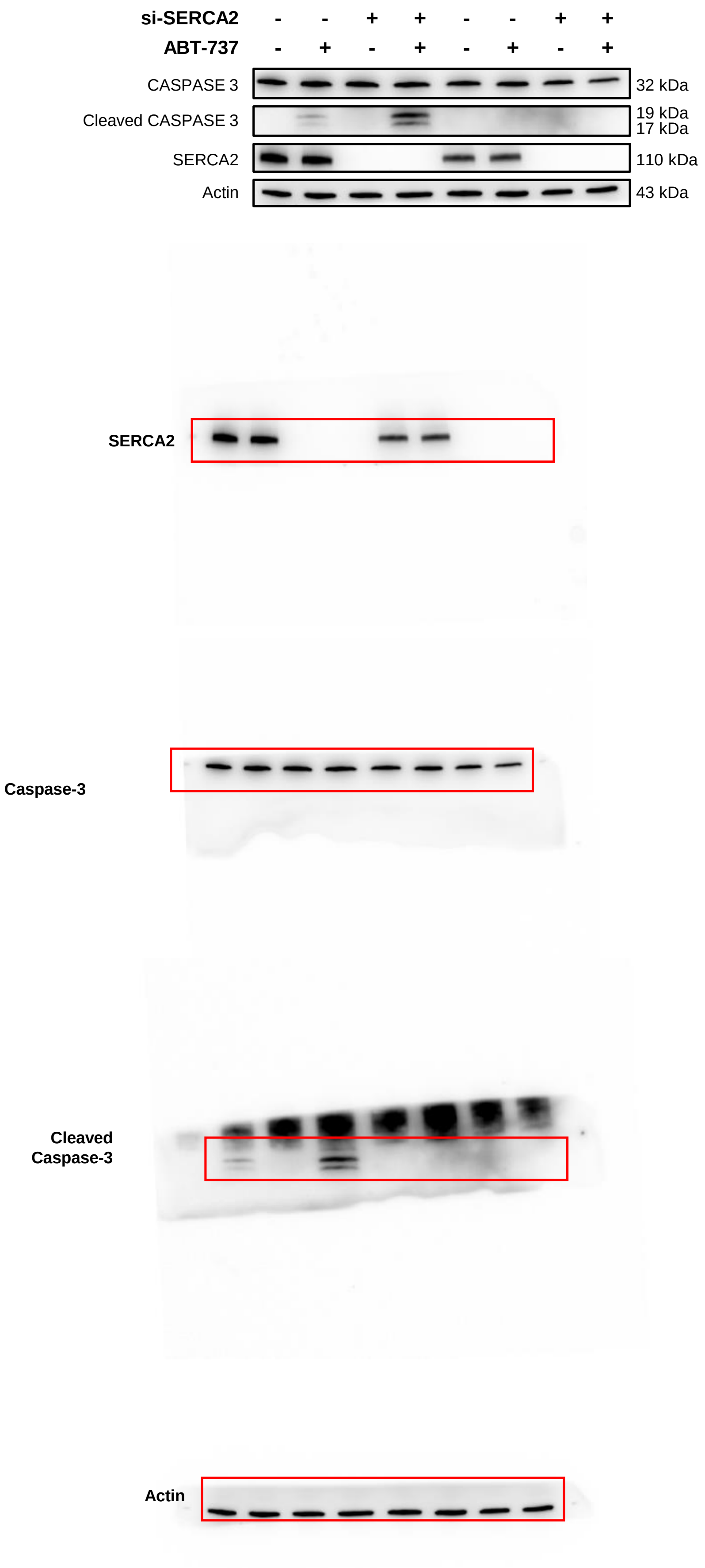
si-SERCA2 - - + + - - + +
ABT-737 - + - + - + - +
CASPASE 3
32 kDa
Cleaved CASPASE 3
19 kDa
17 kDa
SERCA2
110 kDa
Actin
43 kDa
SERCA2
Caspase-3
Cleaved
Caspase-3
Actin

Figures 2E

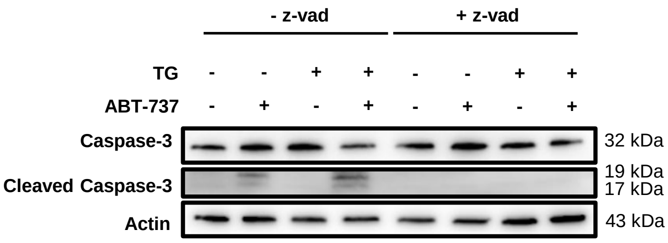
- z-vad
+ z-vad
TG - - + + - - + +
ABT-737 - + - + - + - +
Caspase-3
Cleaved Caspase-3
Actin
32 kDa
19 kDa
17 kDa
43 kDa

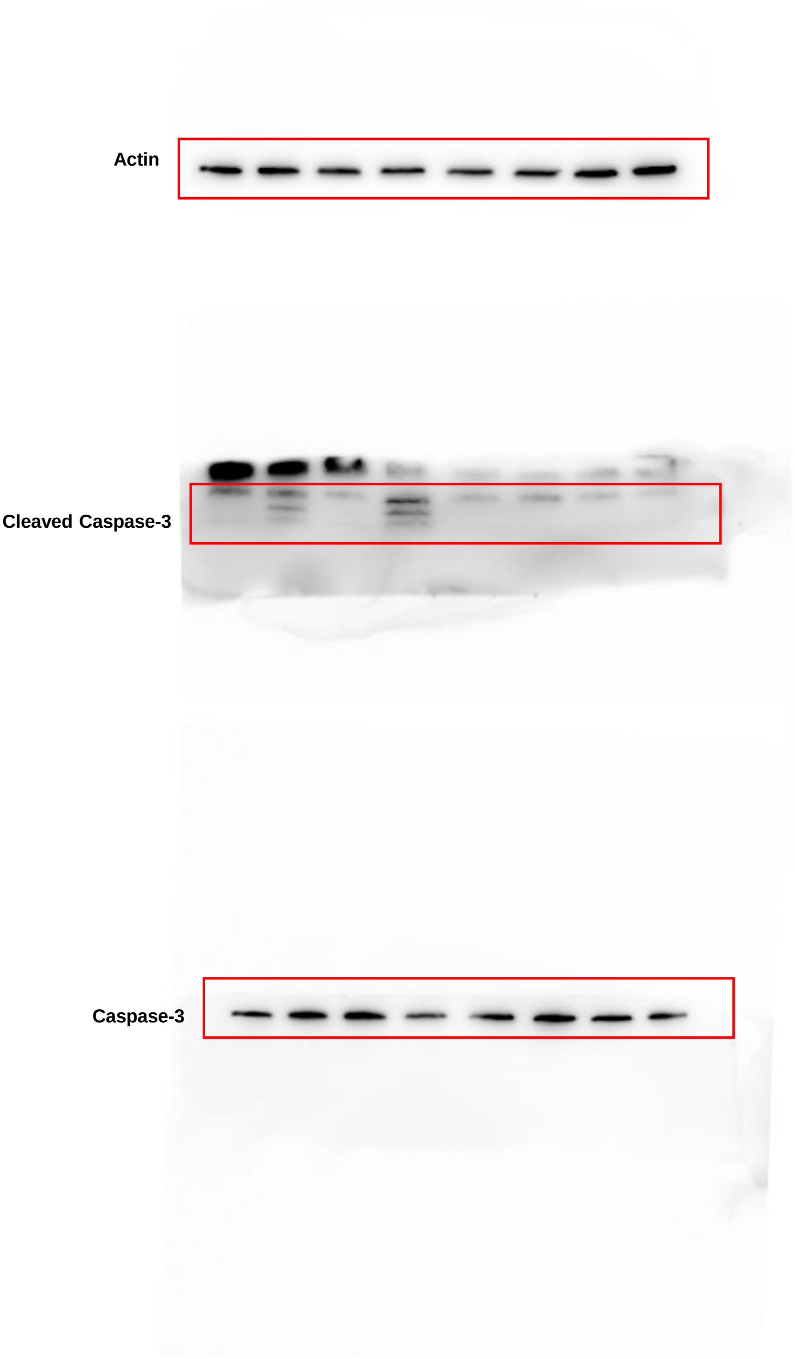
Actin
Cleaved Caspase-3
Caspase-3

Figures 3A

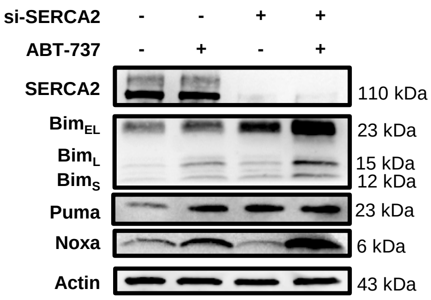

Bim$_{EL}$
Bim$_{L}$
Bim$_{S}$

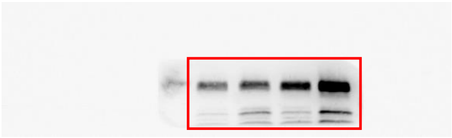

Puma

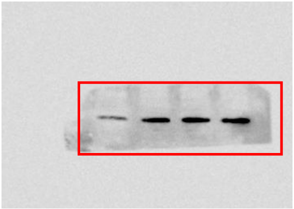

Noxa

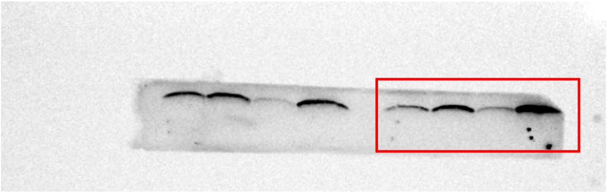

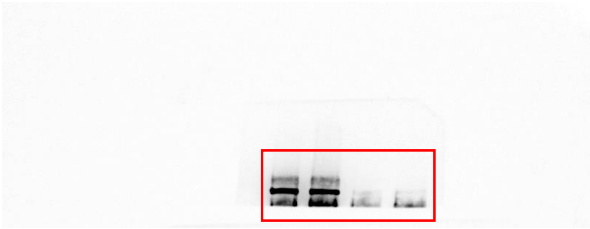

SERCA2

Actin

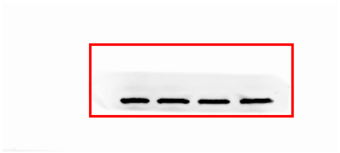

Figures 3A

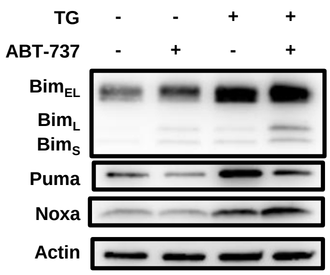
TG - - + +
ABT-737 - + - +
Bim_{EL}
Bim_L
Bim_S
Puma
Noxa
Actin

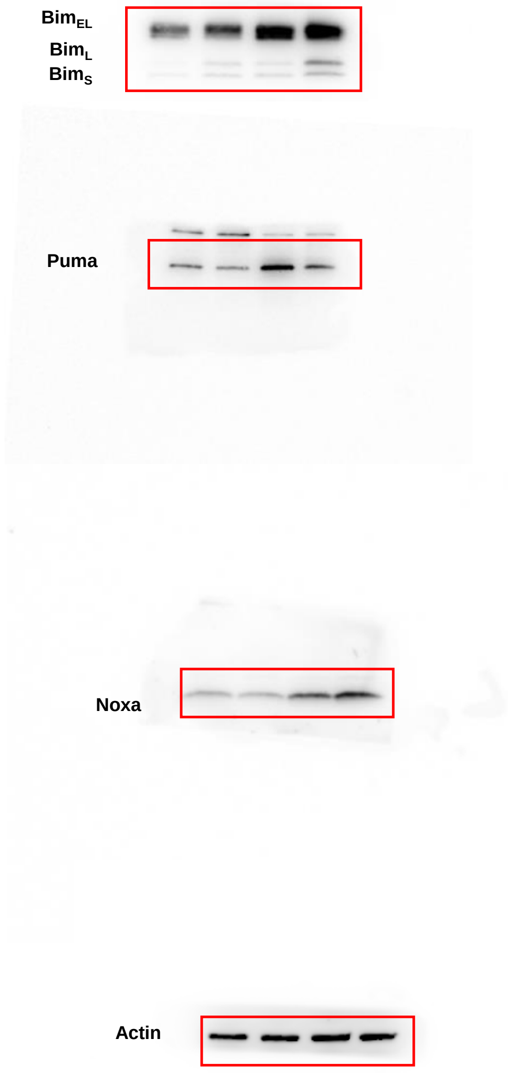
Bim_{EL}
Bim_L
Bim_S
Puma
Noxa
Actin

Figures 3D

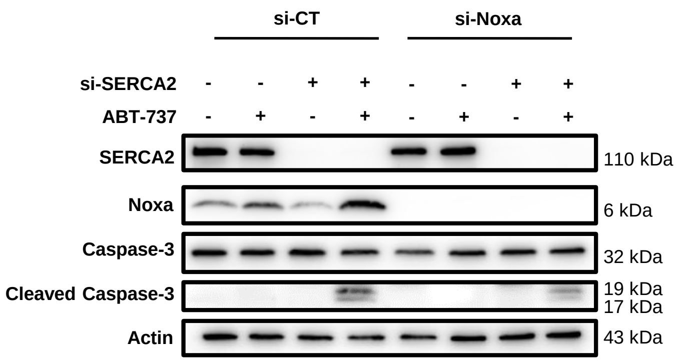
si-CT
si-Noxa
si-SERCA2 - - + + - - + +
ABT-737 - + - + - + - +
SERCA2
110 kDa
Noxa
6 kDa
Caspase-3
32 kDa
Cleaved Caspase-3
19 kDa
17 kDa
Actin
43 kDa

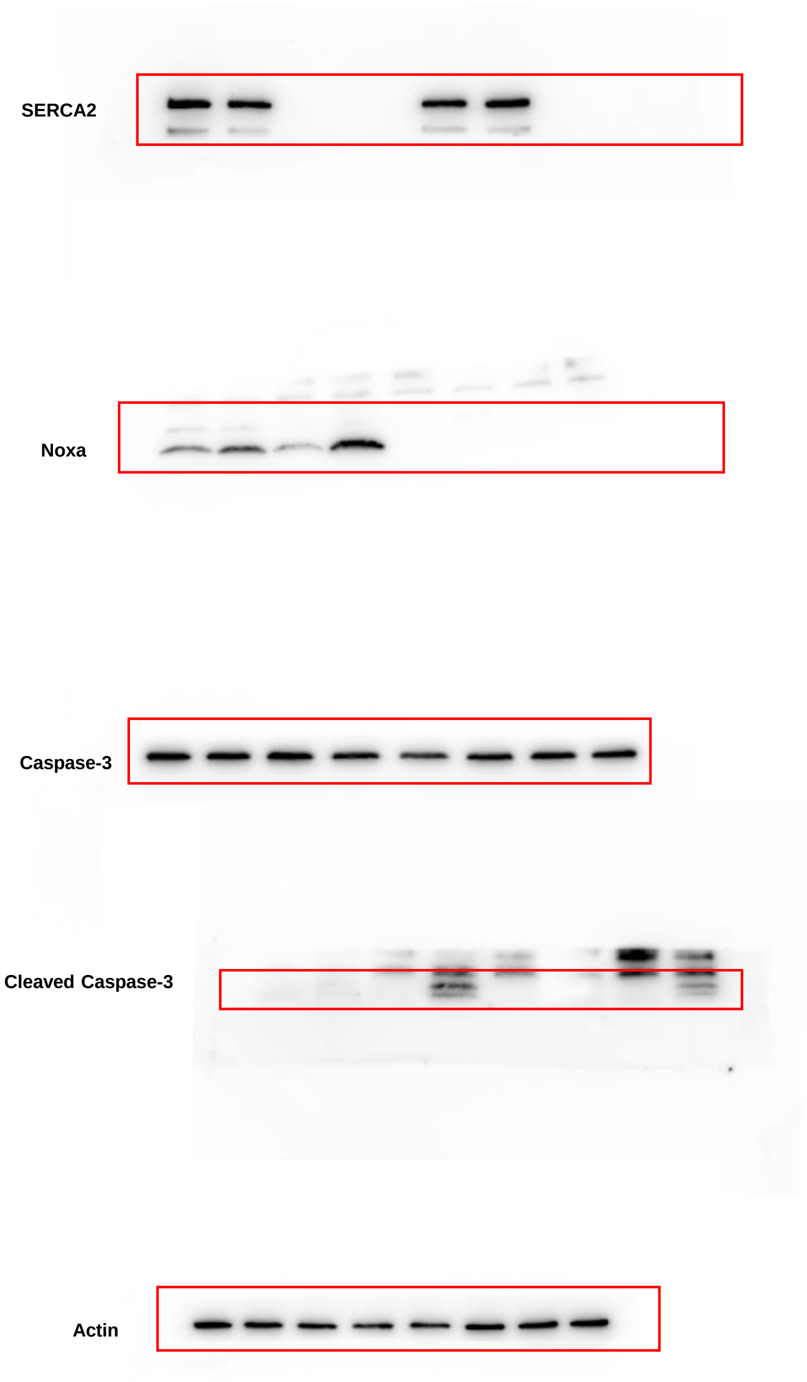
SERCA2
Noxa
Caspase-3
Cleaved Caspase-3
Actin

Figures 3D

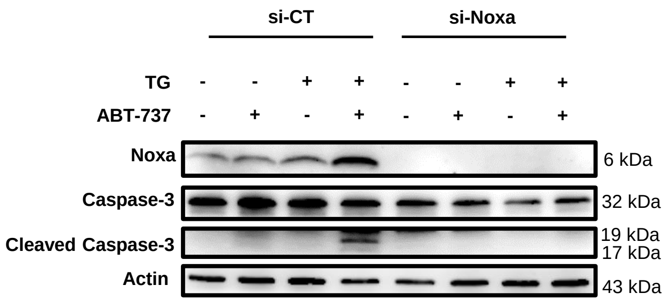
si-CT
si-Noxa
TG - - + + - - + +
ABT-737 - + - + - + - +
Noxa
Caspase-3
Cleaved Caspase-3
Actin
6 kDa
32 kDa
19 kDa
17 kDa
43 kDa

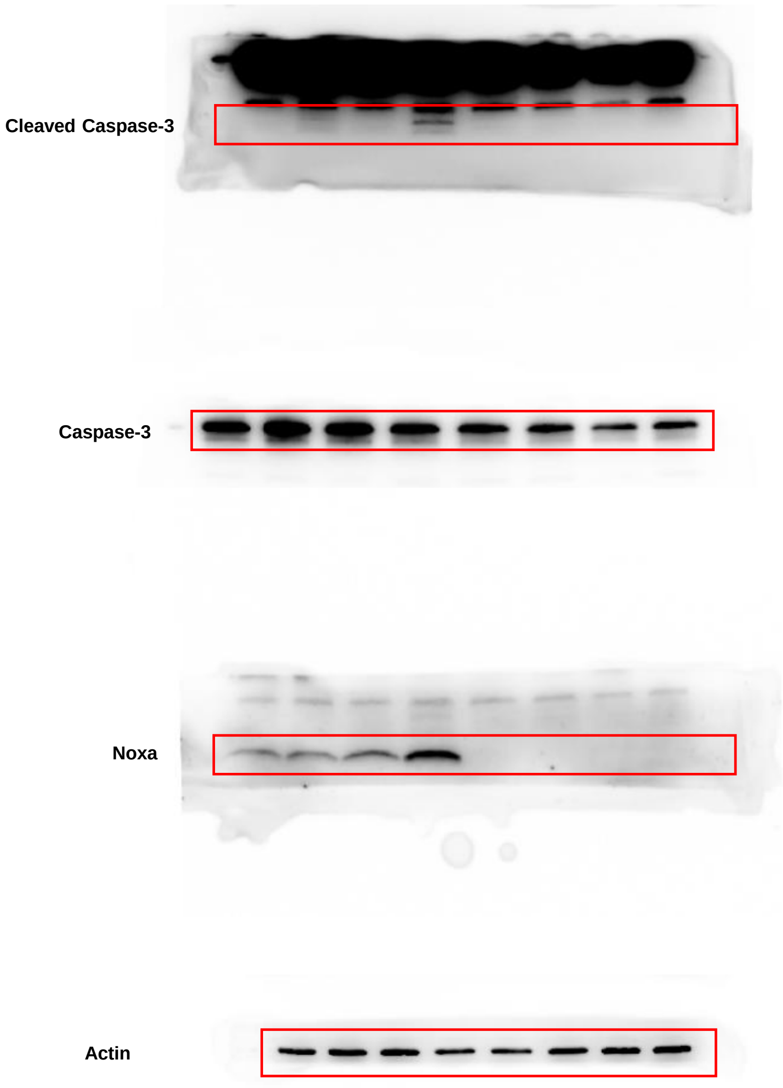
Cleaved Caspase-3
Caspase-3
Noxa
Actin

Figures 4B

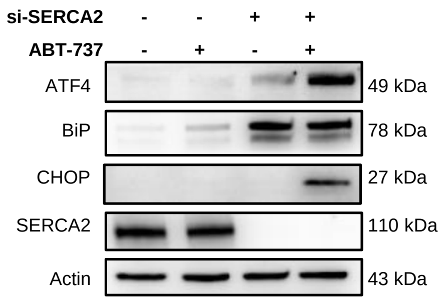

ATF4

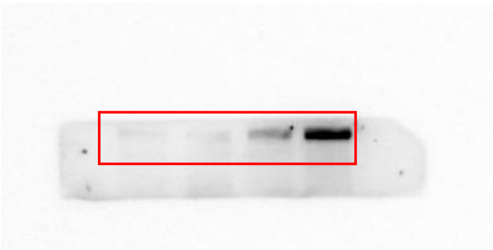

SERCA2

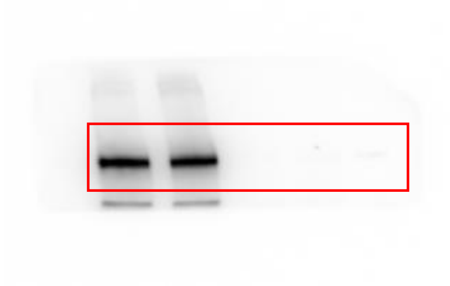

BiP

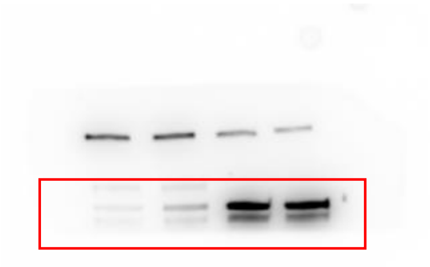

Actin

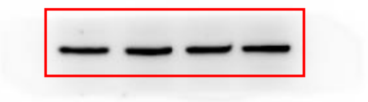

CHOP

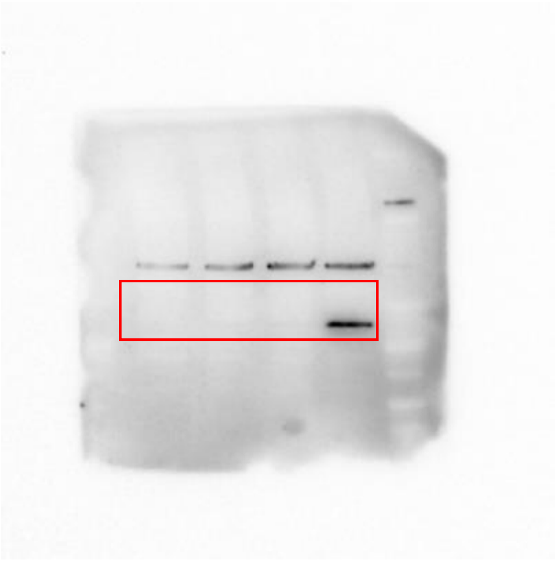

Figures 4B

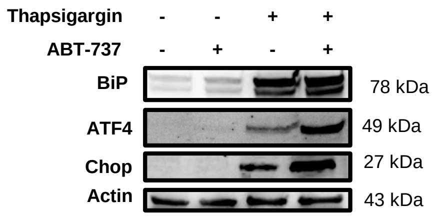

BiP

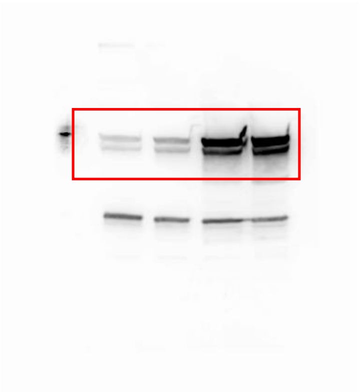

ATF4

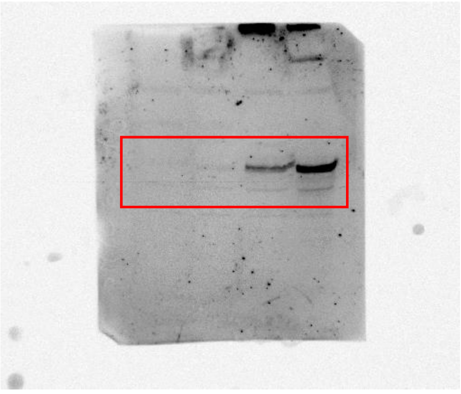

Actine

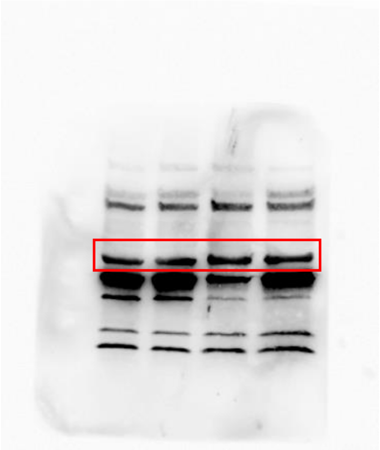

CHOP

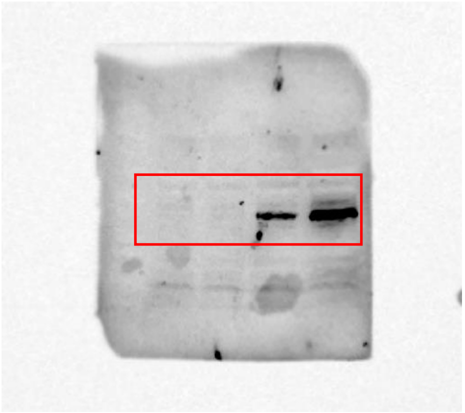

Figures 4E

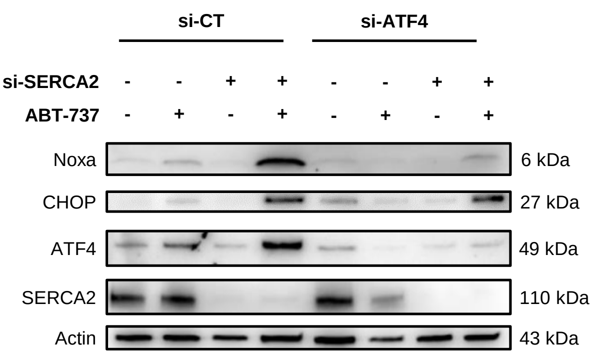

Noxa

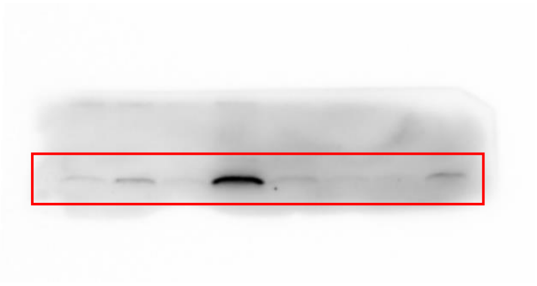

CHOP

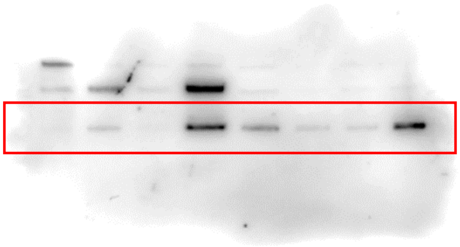

ATF4

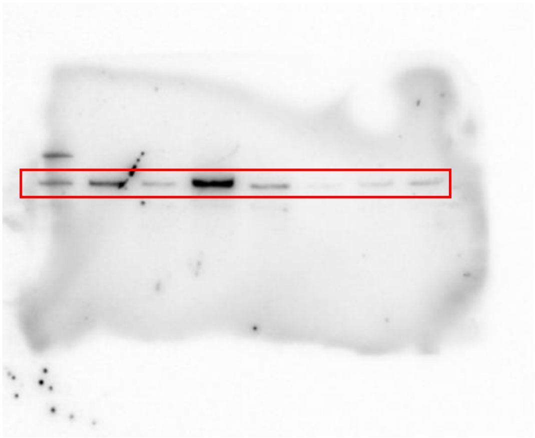

SERCA2

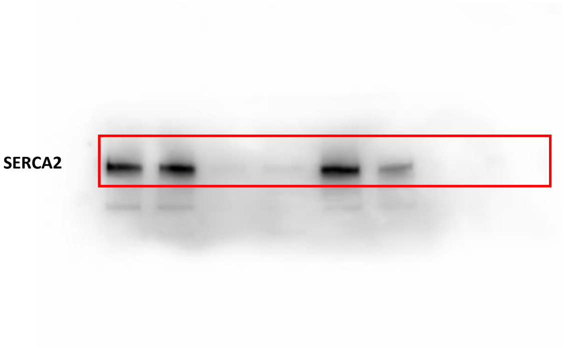

Actin

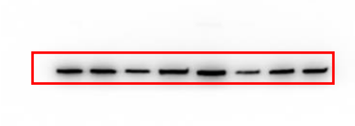

Figures 4E

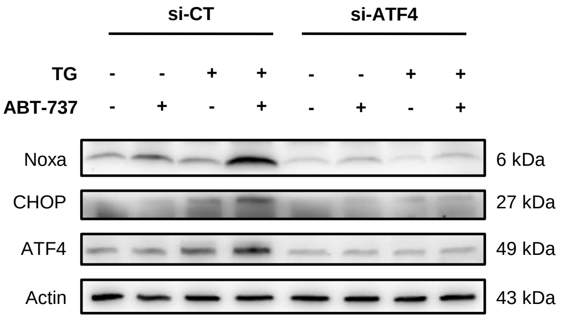

Noxa

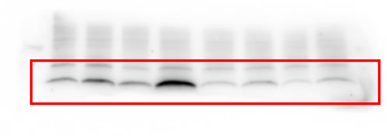

ATF4

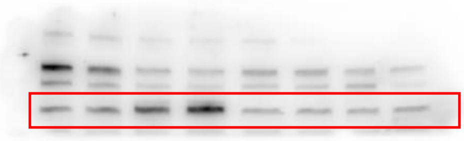

CHOP

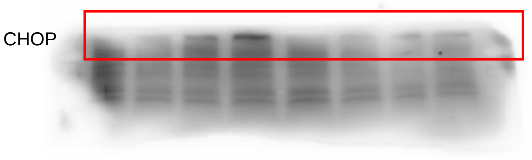

Actin

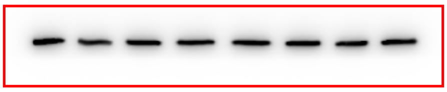

Figures 5C

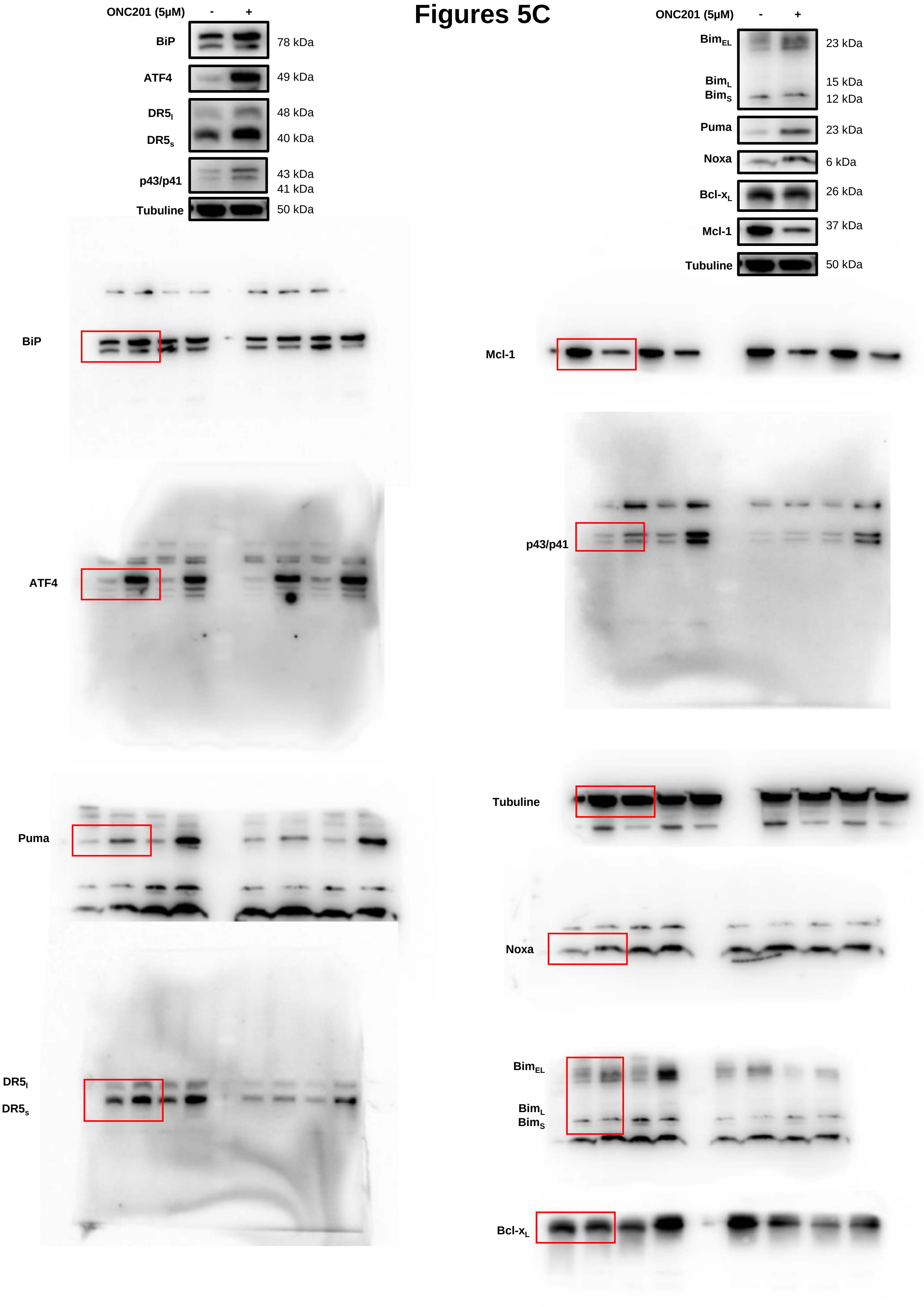

ONC201 (5µM) - +
BiP 78 kDa
ATF4 49 kDa
DR5l 48 kDa
DR5s 40 kDa
p43/p41 43 kDa 41 kDa
Tubuline 50 kDa
ONC201 (5µM) - +
BimEL 23 kDa
BimL 15 kDa
BimS 12 kDa
Puma 23 kDa
Noxa 6 kDa
Bcl-xL 26 kDa
Mcl-1 37 kDa
Tubuline 50 kDa
BiP
ATF4
Puma
DR5l
DR5s
Mcl-1
p43/p41
Tubuline
Noxa
BimEL
BimL
BimS
Bcl-xL

Figures 5E

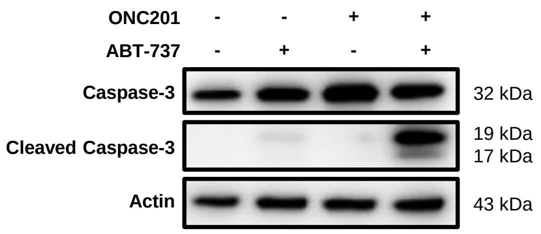
ONC201 - - + +
ABT-737 - + - +
Caspase-3
32 kDa
Cleaved Caspase-3
19 kDa
17 kDa
Actin
43 kDa

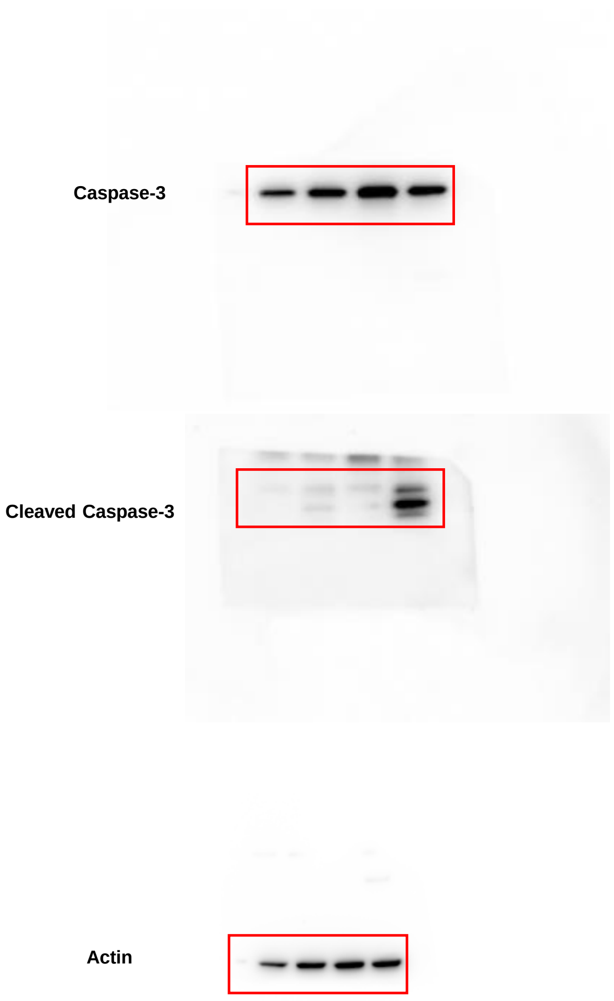
Caspase-3
Cleaved Caspase-3
Actin

Figures 5G

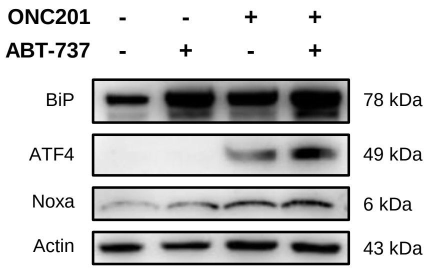
ONC201 - - + +
ABT-737 - + - +
BiP
78 kDa
ATF4
49 kDa
Noxa
6 kDa
Actin
43 kDa

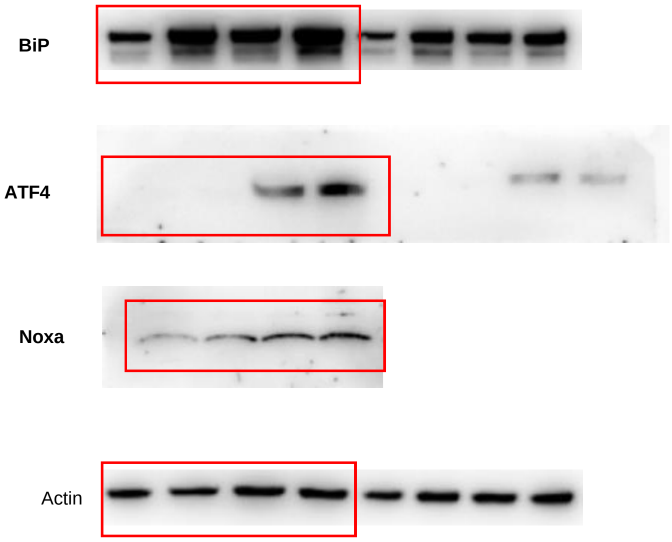
BiP
ATF4
Noxa
Actin

Figures 5J

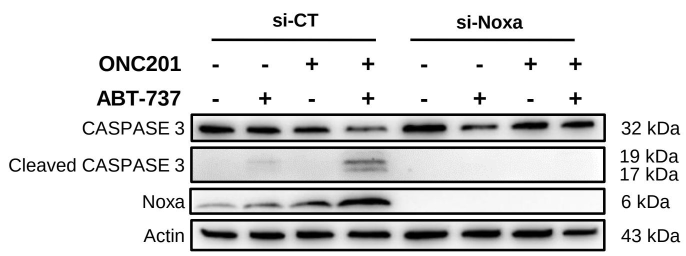
si-CT
si-Noxa
ONC201 - - + + - - + +
ABT-737 - + - + - + - +
CASPASE 3
Cleaved CASPASE 3
Noxa
Actin
32 kDa
19 kDa
17 kDa
6 kDa
43 kDa

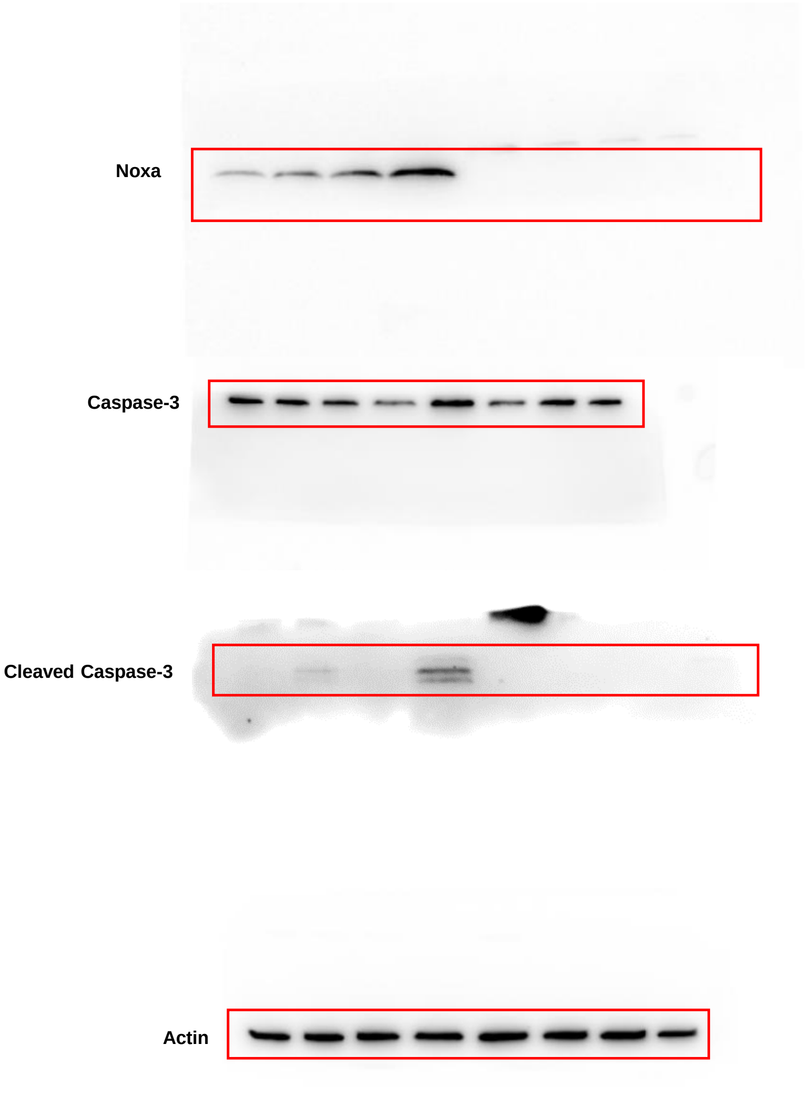
Noxa
Caspase-3
Cleaved Caspase-3
Actin

Figures 5K

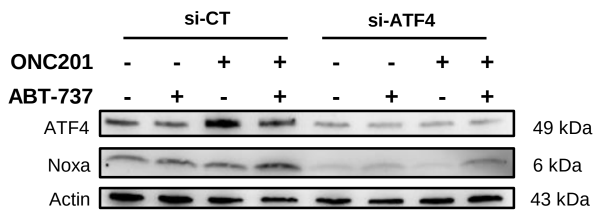
si-CT
si-ATF4
ONC201 - - + + - - + +
ABT-737 - + - + - + - +
ATF4
Noxa
Actin
49 kDa
6 kDa
43 kDa

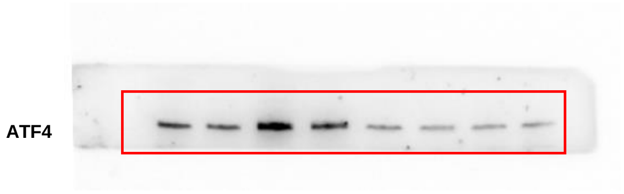
ATF4

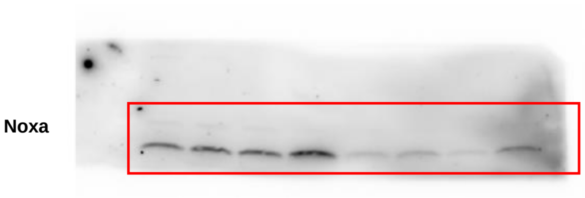
Noxa

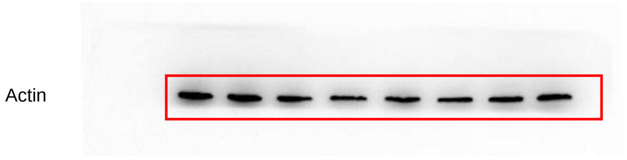
Actin

Figures 6C

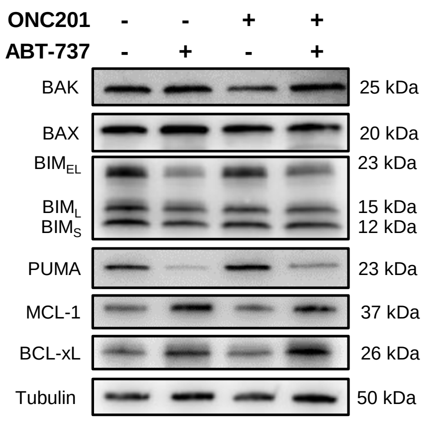
ONC201 - - + +
ABT-737 - + - +
BAK
25 kDa
BAX
20 kDa
BIM$_{EL}$
23 kDa
BIM$_L$
BIM$_S$
15 kDa
12 kDa
PUMA
23 kDa
MCL-1
37 kDa
BCL-xL
26 kDa
Tubulin
50 kDa

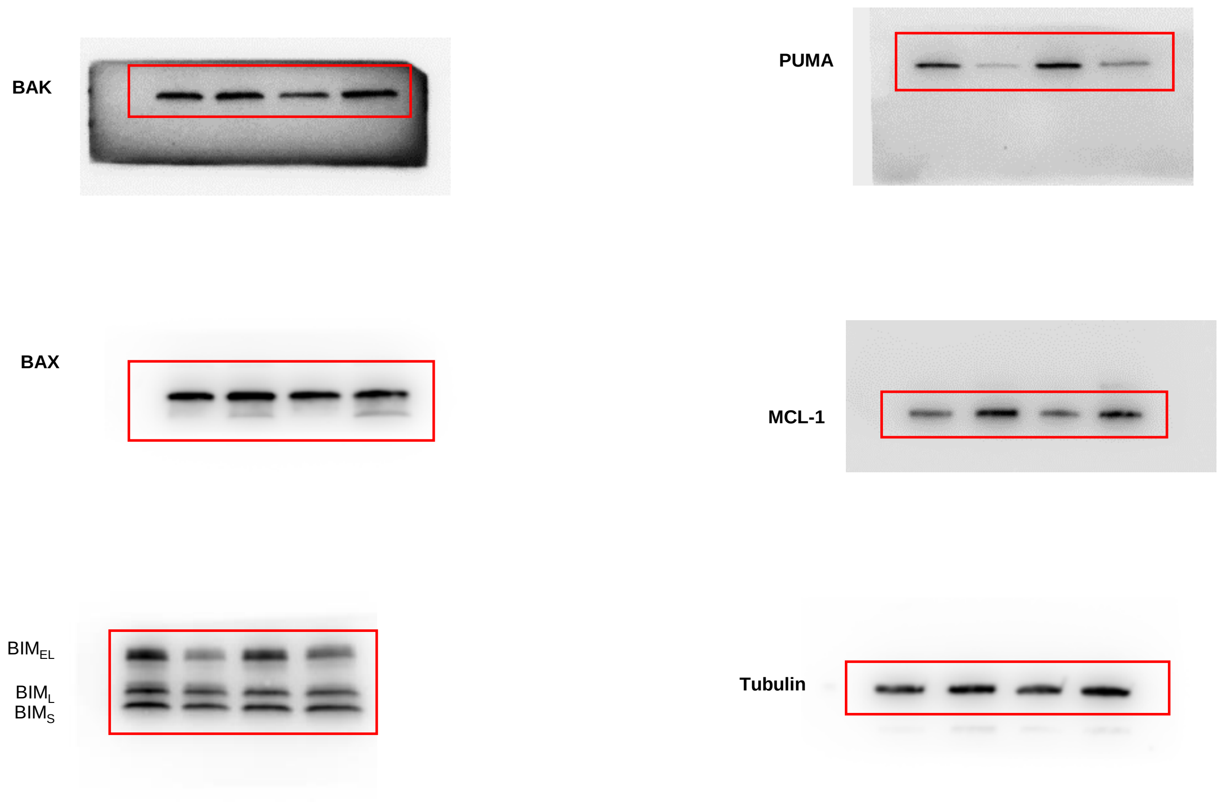
BAK
PUMA
BAX
MCL-1
BIM$_{EL}$
BIM$_L$
BIM$_S$
Tubulin

Figures 6C

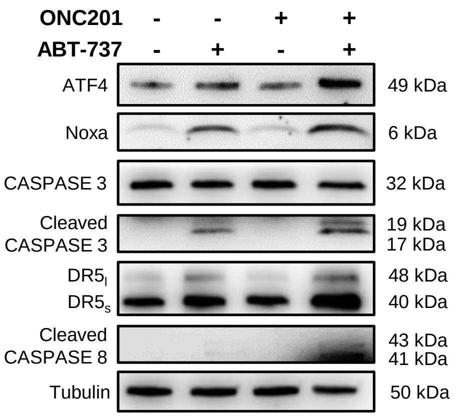
ONC201 - - + +
ABT-737 - + - +
ATF4
49 kDa
Noxa
6 kDa
CASPASE 3
32 kDa
Cleaved CASPASE 3
19 kDa
17 kDa
$DR5_l$
$DR5_s$
48 kDa
40 kDa
Cleaved CASPASE 8
43 kDa
41 kDa
Tubulin
50 kDa

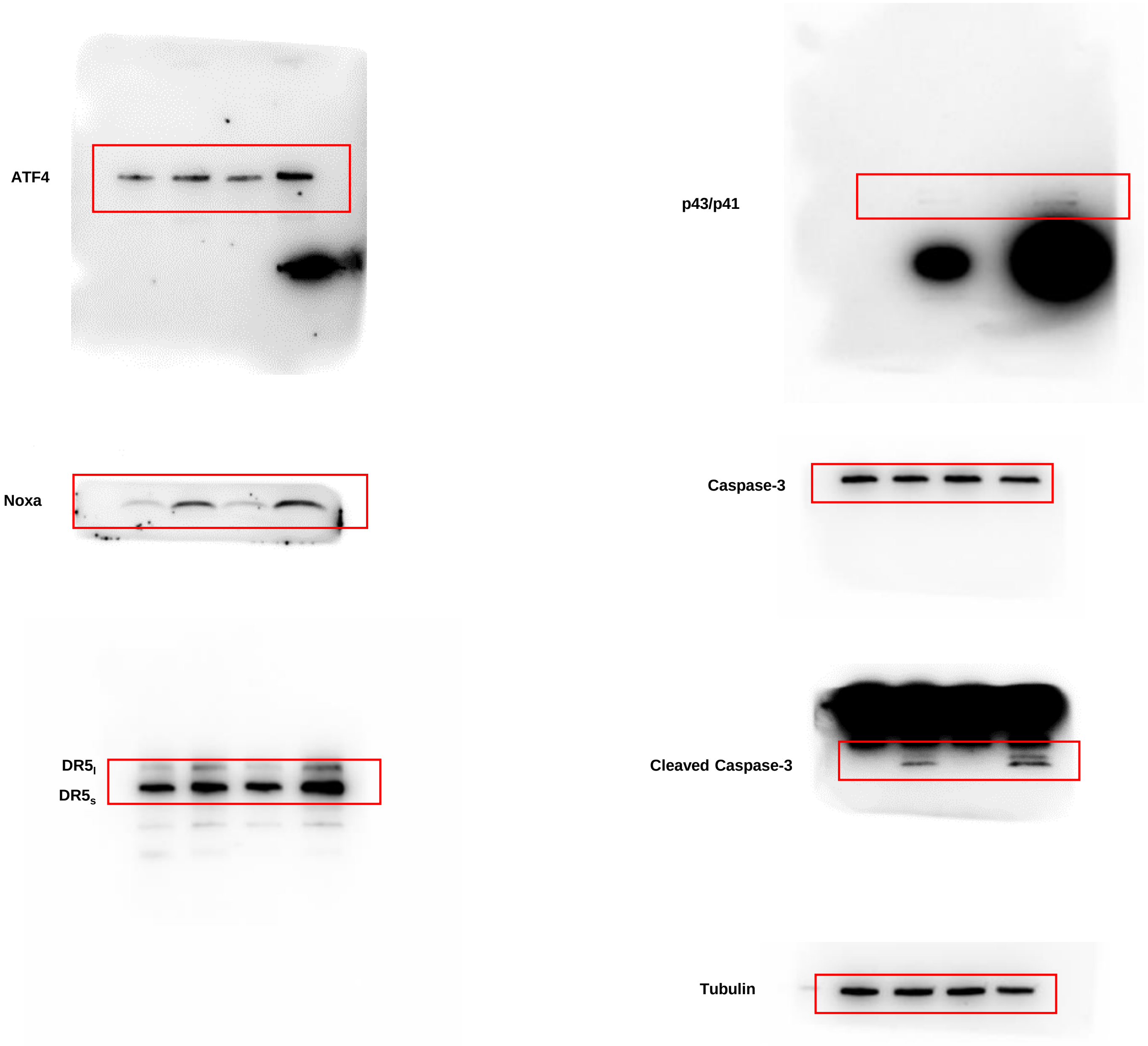
ATF4
p43/p41
Noxa
Caspase-3
Cleaved Caspase-3
$DR5_l$
$DR5_s$
Tubulin

Supplementary Figure 1B

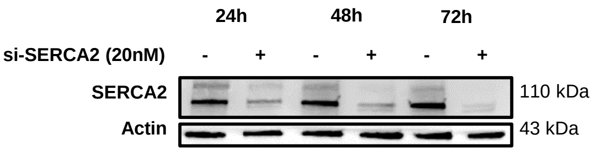

SERCA2

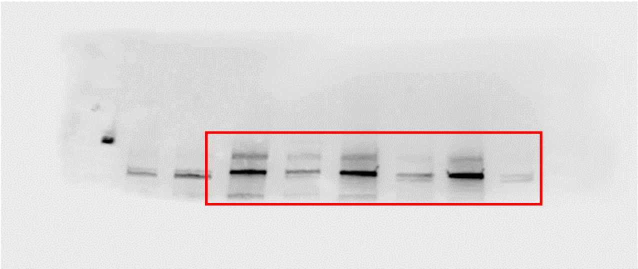

Actin

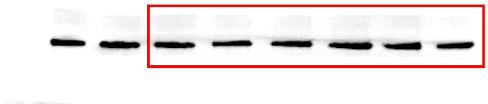

Supplementary Figure 2A

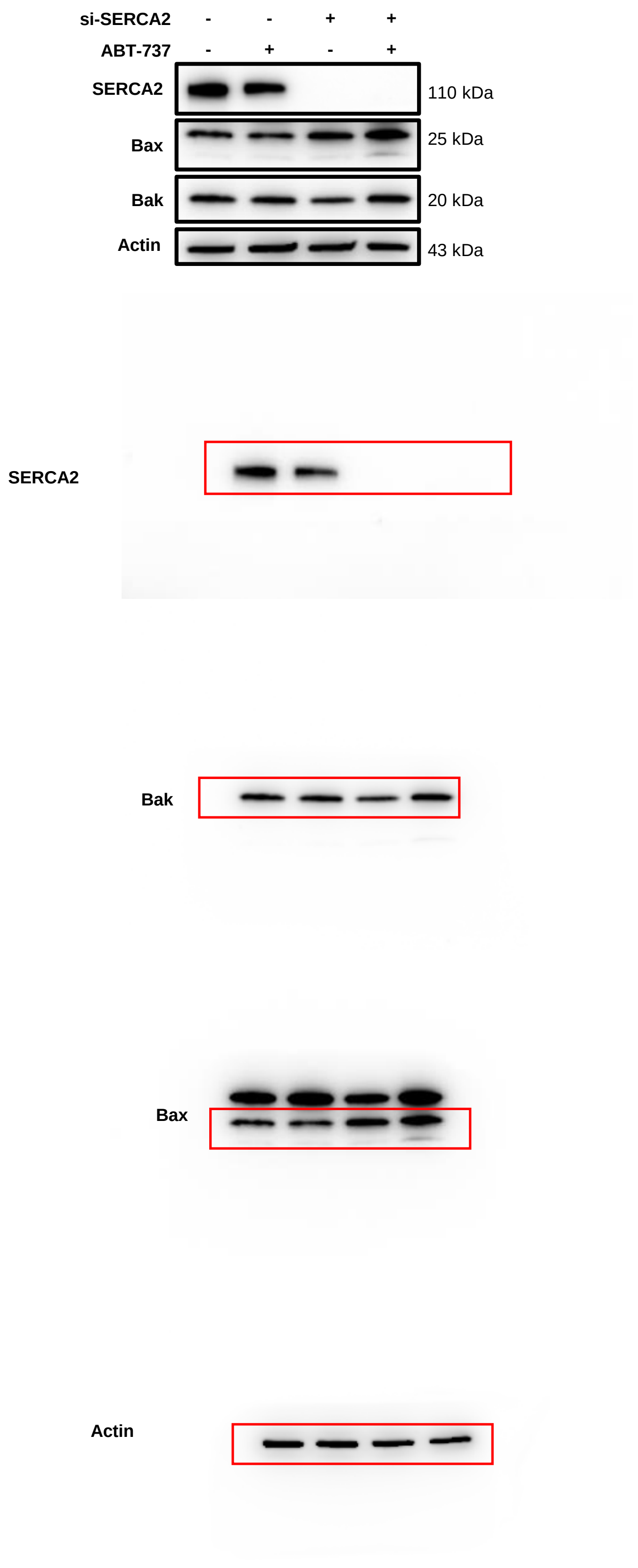

Supplementary Figure 2A

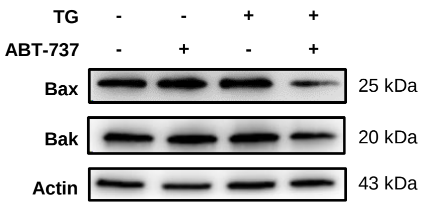

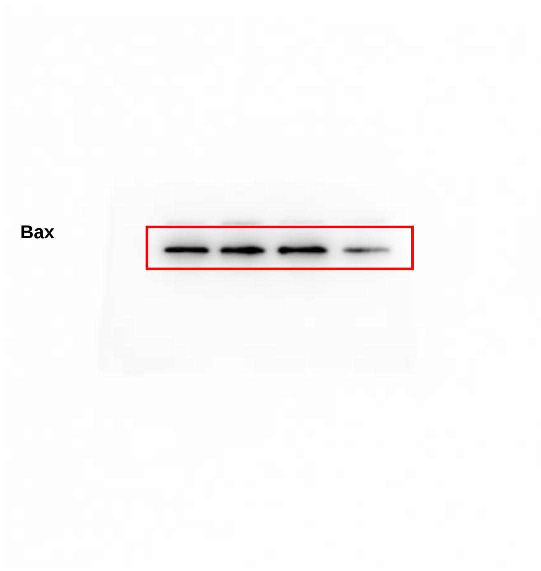

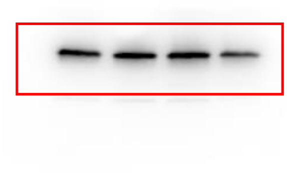

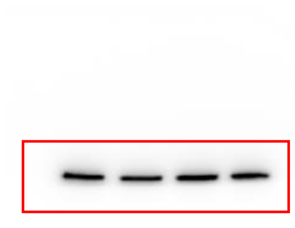

Supplementary Figure 2D

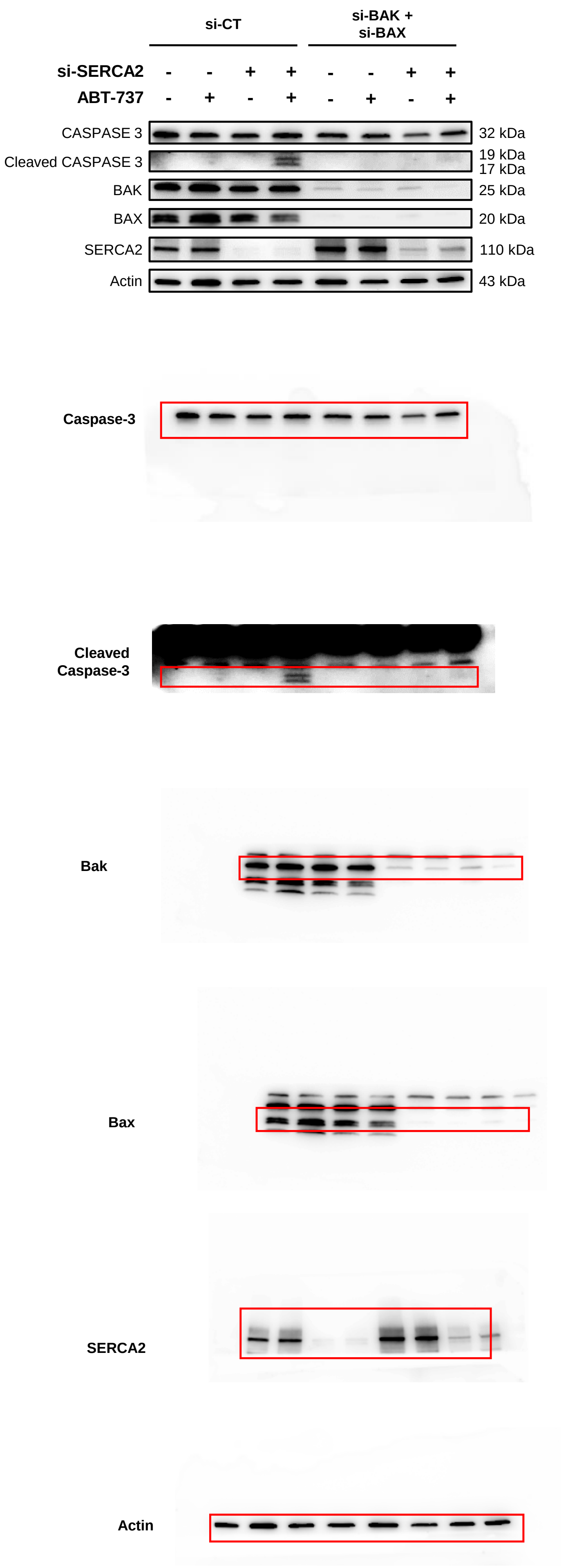

Supplementary Figure 2D

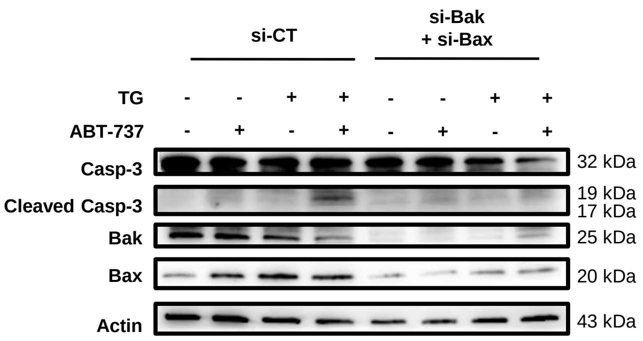

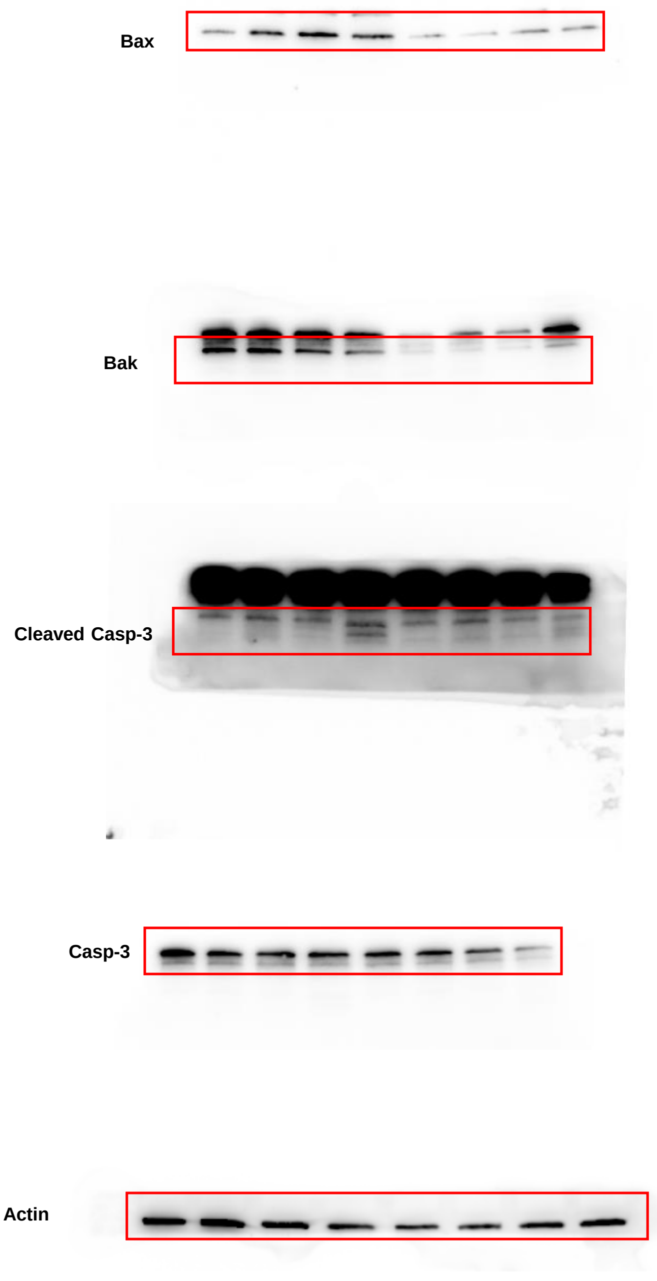

Supplementary Figure 4B

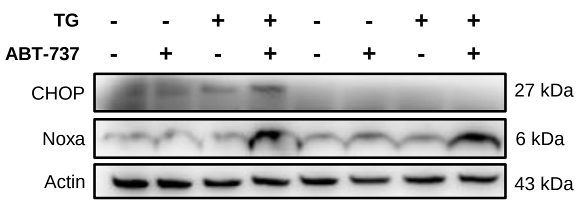

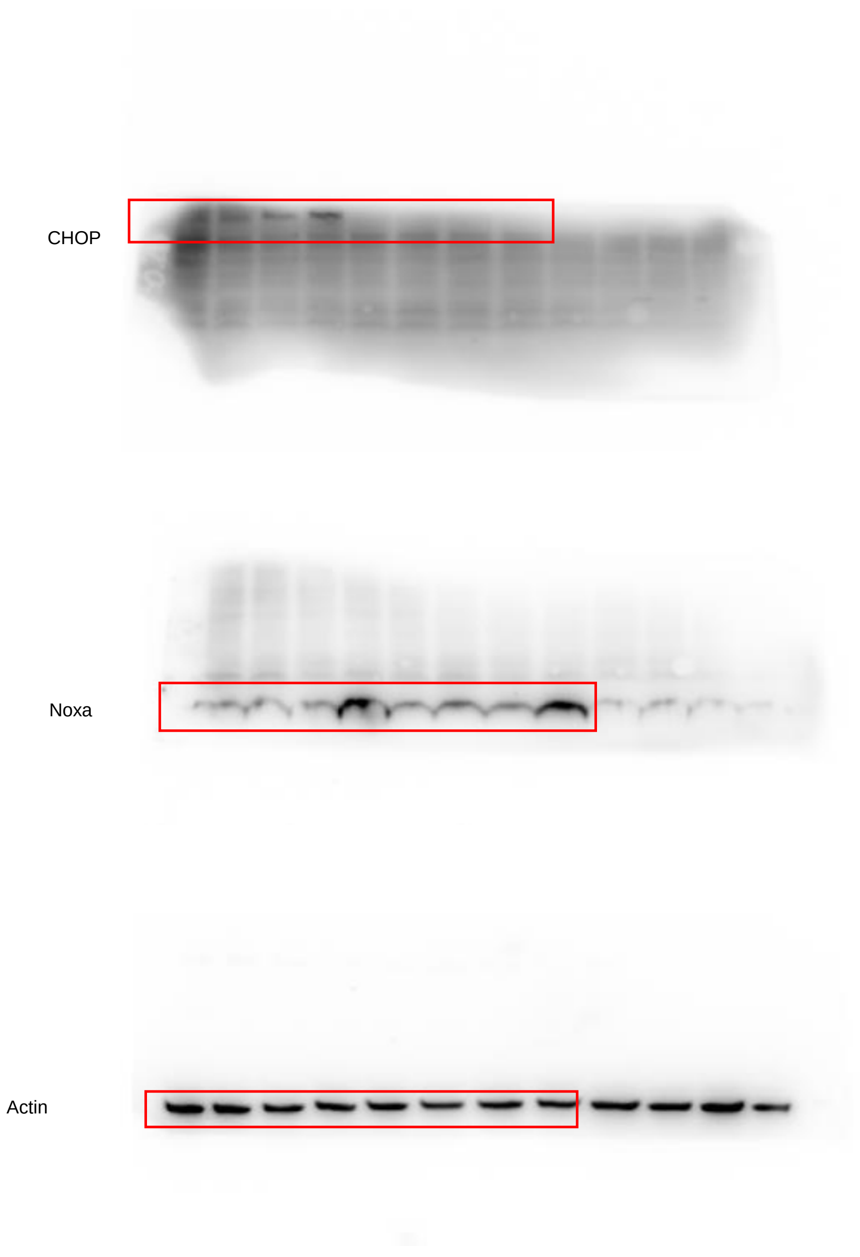

Supplementary Figure S5A

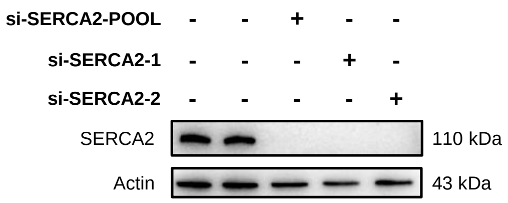

SERCA2

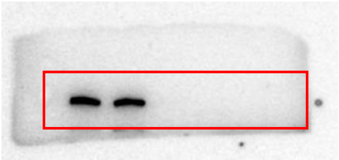

Actin

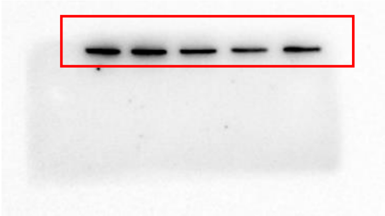

Supplementary Figure S5B

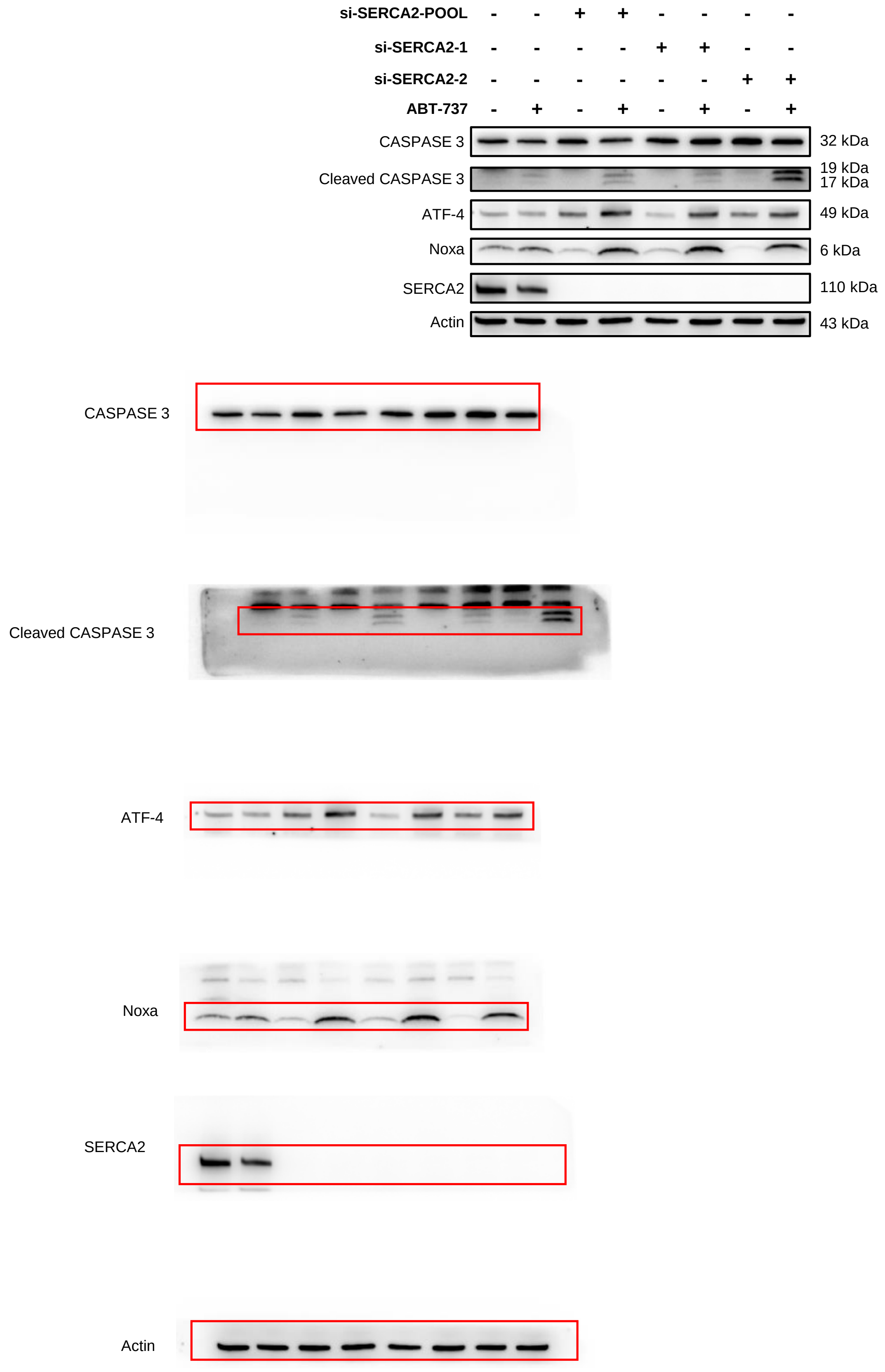

Supplementary Figure S6A

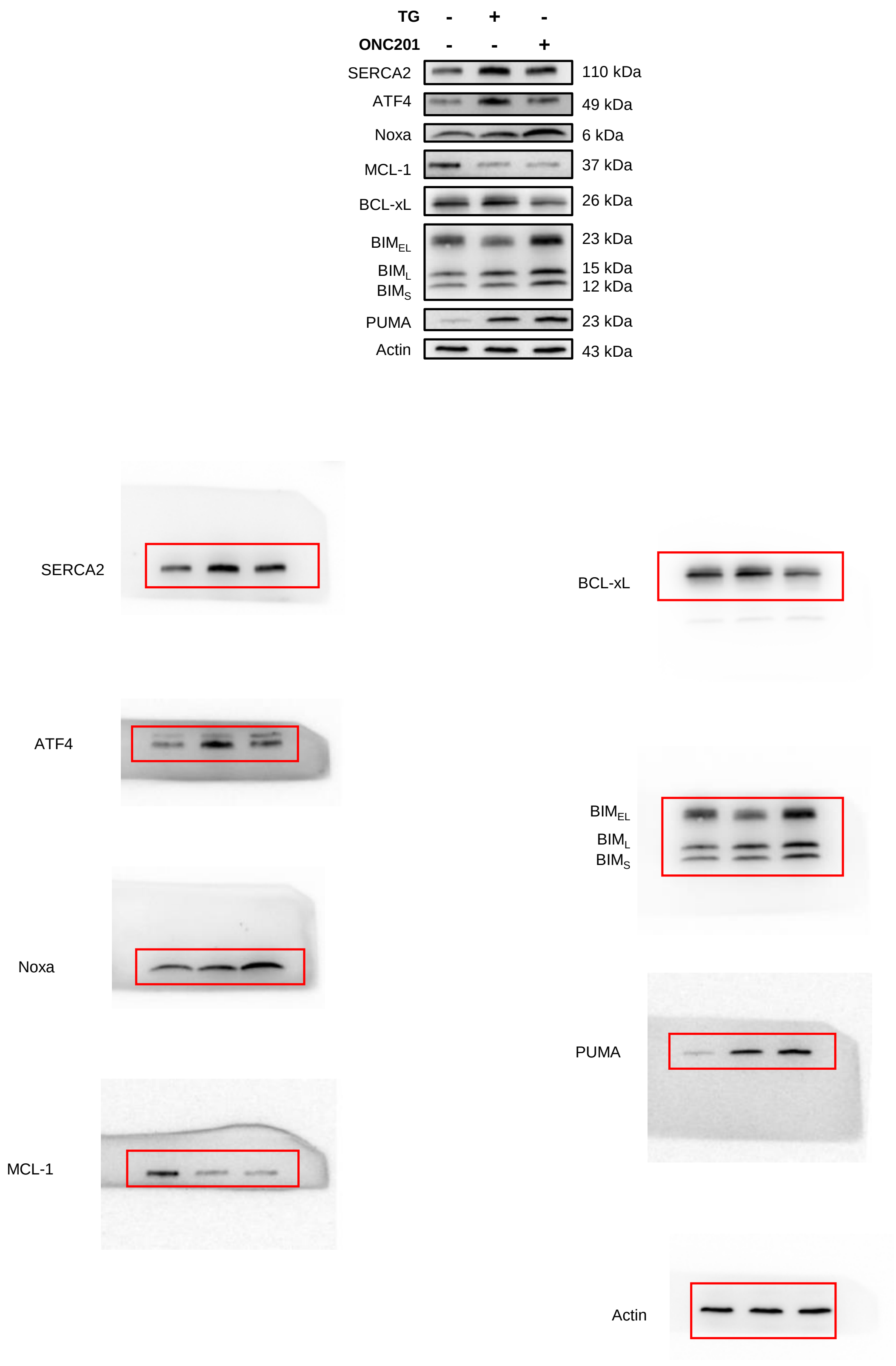

Supplementary Figure S6D

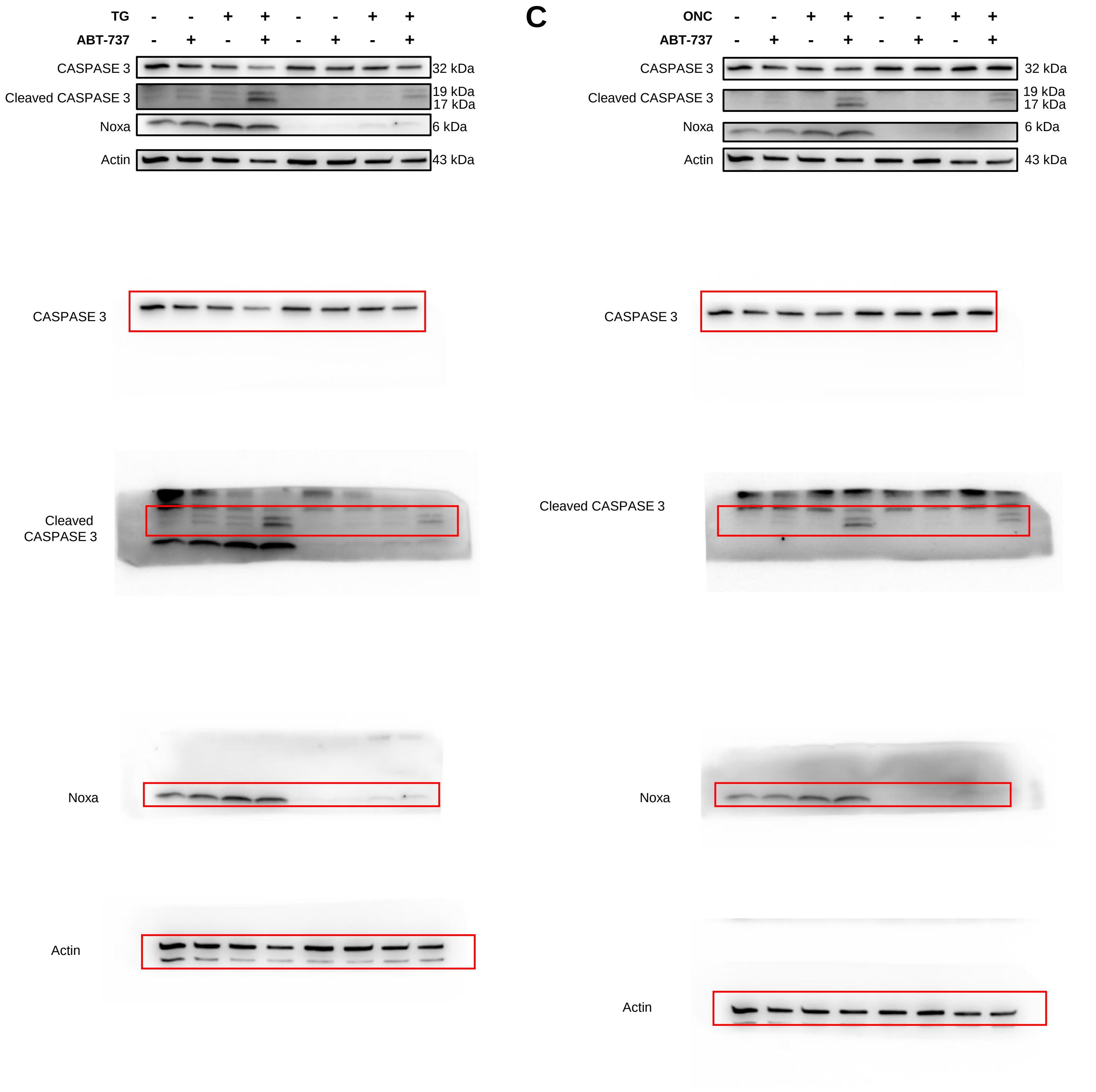

Supplementary Figure S6E

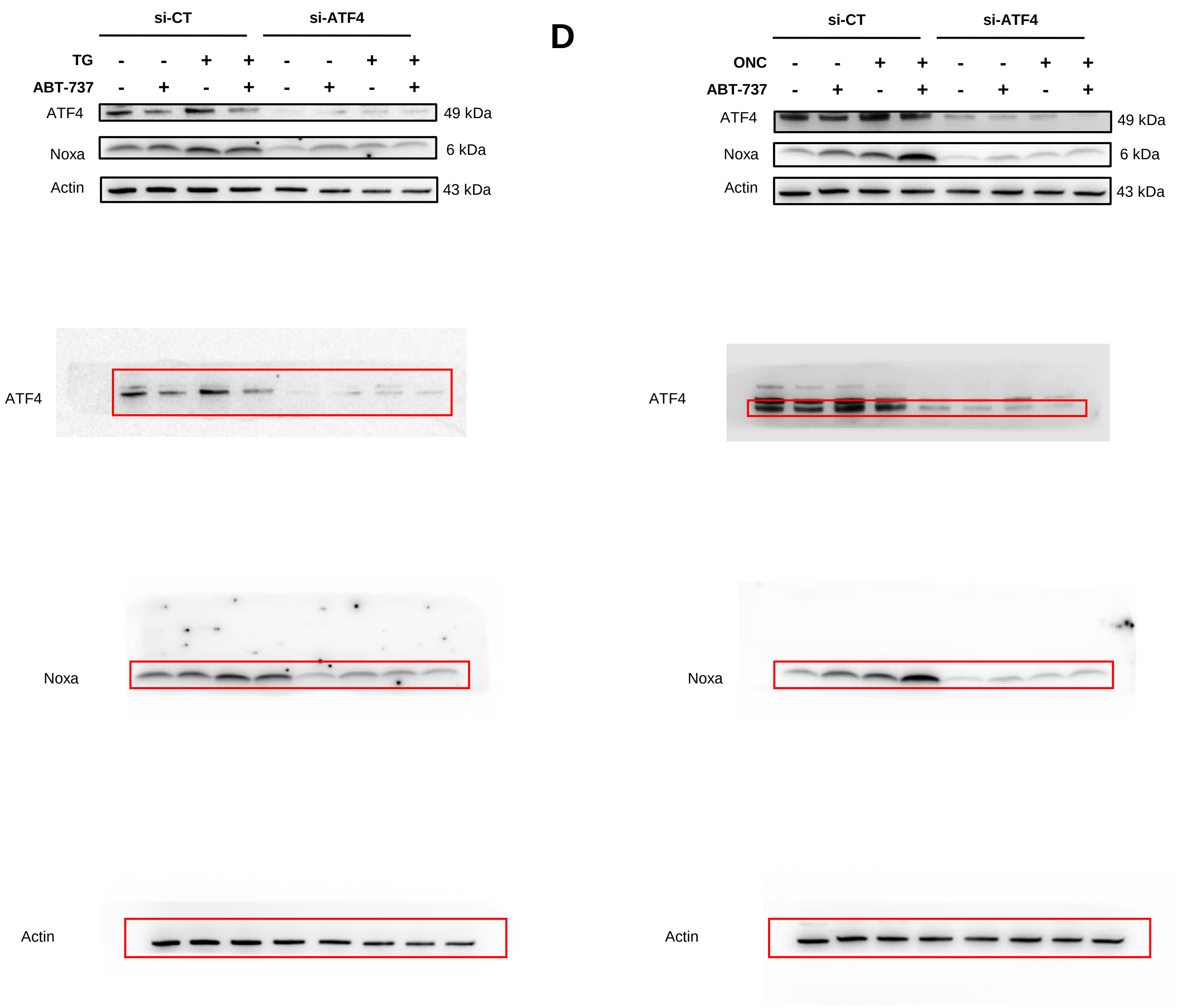

Supplement: Supplementary file 7 — Original Western Blots [file 41419_2026_8559_MOESM7_ESM.pdf]
